# Genetic architecture behind developmental and seasonal control of tree growth and wood properties in Norway spruce

**DOI:** 10.3389/fpls.2022.927673

**Published:** 2022-08-09

**Authors:** Zhi-Qiang Chen, Yanjun Zan, Linghua Zhou, Bo Karlsson, Hannele Tuominen, Maria Rosario García-Gil, Harry X. Wu

**Affiliations:** ^1^Department Forest Genetics and Plant Physiology, Umeå Plant Science Centre, Swedish University of Agricultural Sciences, Umeå, Sweden; ^2^Skogforsk, Ekebo, Svalöv, Sweden; ^3^The Commonwealth Scientific and Industrial Research Organisation (CSIRO) National Collection Research Australia, Black Mountain Laboratory, Canberra, ACT, Australia

**Keywords:** wood properties, seasonal variation, developmental stage, genome-wide association, Norway spruce

## Abstract

Genetic control of tree growth and wood formation varies depending on the age of the tree and the time of the year. Single-locus, multi-locus, and multi-trait genome-wide association studies (GWAS) were conducted on 34 growth and wood property traits in 1,303 Norway spruce individuals using exome capture to cover ~130K single-nucleotide polymorphisms (SNPs). GWAS identified associations to the different wood traits in a total of 85 gene models, and several of these were validated in a progenitor population. A multi-locus GWAS model identified more SNPs associated with the studied traits than single-locus or multivariate models. Changes in tree age and annual season influenced the genetic architecture of growth and wood properties in unique ways, manifested by non-overlapping SNP loci. In addition to completely novel candidate genes, SNPs were located in genes previously associated with wood formation, such as cellulose synthases and a NAC transcription factor, but that have not been earlier linked to seasonal or age-dependent regulation of wood properties. Interestingly, SNPs associated with the width of the year rings were identified in homologs of *Arabidopsis thaliana* BARELY ANY MERISTEM 1 and rice BIG GRAIN 1, which have been previously shown to control cell division and biomass production. The results provide tools for future Norway spruce breeding and functional studies.

## Introduction

Forest trees, particularly conifers, produce wood with distinct properties depending on their developmental stage (age) and the annual season (Li et al., 2011). At young ages, conifer trees typically produce so-called juvenile wood (JW) that has lower wood density and stiffness than mature wood (MW) formed at older ages (Zobel and Sprague, 1998). The seasonal variation, manifested by the formation of earlywood (EW), transition wood (TW), and latewood (LW), results in drastic changes in wood density, cell size, and cell wall thickness (Olsson et al., 1998; Park and Spiecker, 2005). Trees in the spring experience fast growth with new needles and little environmental stress except for short cold snaps, while trees in the autumn usually experience slow growth associated with drought and short days, with wood cells becoming smaller, but with thicker cell walls.

Conifer breeding has traditionally focused on growth traits (Wu et al., 2007; Isik and McKeand, 2019), resulting often in unfavorable effects on wood quality (Bouffier et al., 2008; Wu et al., 2008) due to the negative genetic correlations between growth rate and wood quality traits, such as wood density (Baltunis et al., 2007; Lenz et al., 2010; Chen et al., 2014; Hong et al., 2014; Hayatgheibi et al., 2017). During the last two decades, considerable efforts have been made to improve wood quality in several conifer species (Isik and Li, 2003; Chen et al., 2015). Genetic variation in wood properties has been quantified for many conifers (Wu et al., 2008), and new breeding strategies including both growth and wood properties have been developed (Wu and Sanchez, 2011; Hallingback et al., 2014). Examples of the novel breeding targets include acceleration of the transition from the JW to MW (Gapare et al., 2006; Hayatgheibi et al., 2018) and improved quality of JW using index selection (Ivković et al., 2010). However, breeding for these properties is hampered by the poor knowledge of the effect of tree age and seasonality on the genetic architecture of growth and wood properties in conifers.

Genome-wide association studies (GWAS) have contributed to substantial advances in human, animal, and plant genetic research (Goddard and Hayes, 2009; Visscher et al., 2017; Mills and Rahal, 2019). However, in conifers, the contribution of GWAS has been more limited due to their large genome size (~15–40 Gb) that challenges the development of a sufficient number of markers (Neale and Kremer, 2011) and the insufficient numbers (commonly <500 individuals) of trees genotyped (Hall et al., 2016; Chen et al., 2021). More recently, several reference genomes and transcriptome assemblies have been made available in tree species, such as Norway spruce (*Picea abies* L. Karst) (Nystedt et al., 2013), loblolly pine (*Pinus taeda* L.) (Neale et al., 2014), white spruce (*Picea glauca*) (Warren et al., 2015), and sugar pine (*Pinus lambertiana* Doug) (Stevens et al., 2016), which now allow GWAS based on exome capture (Vidalis et al., 2018), genotyping-by-sequencing (GBS) (Pan et al., 2015), SNP arrays from transcripts (Howe et al., 2020), and re-sequencing (Wang et al., 2018; De La Torre et al., 2019; Bernhardsson et al., 2021).

Norway spruce (*Picea abies*) is the most important commercial tree species in Northern Europe. GWAS has been conducted for several wood properties using 517 Norway spruce individuals (Baison et al., 2019, 2020). SNPs have been identified for the traits of wood density, stiffness, the number of cells, and cell wall thickness. However, the genetic architecture of wood traits measured at different seasons and ages has not been systematically investigated by GWAS for Norway spruce or any other forest tree species (Beaulieu et al., 2011). In this study, we implemented GWAS to study how tree age and seasonal variation influence the genetic architecture of growth and wood traits in 1,303 trees of Norway spruce. The accuracy of the GWAS analyses was the highest possible due to sufficient SNP coverage and the availability of improved genome annotation. The GWAS revealed genes that have earlier been implicated in the seasonal and developmental control of growth and wood properties but also novel genes that putatively shape the seasonal and developmental changes in wood formation. In addition, we examined whether multi-locus and multi-trait models could improve the statistical accuracy of GWAS.

## Materials and methods

### Plant materials

Two large progeny trials, locally called Höreda (57° 37′N and 15° 00′E) and Maltesholm (55° 53′N and 13° 55′E), were established in 1990 in southern Sweden, using 1,375 open-pollinated families (Chen et al., 2015). A randomized incomplete block design with single-tree plots was used in the two trials. Details on the field design, soil type, and climate condition can be found in Chen et al. (2014). A total of 1,303 wood samples, one tree per family, were taken in two different years from the Höreda trial. The first batch of 505 increment cores (12 mm) was sampled in 2010 as part of an earlier project (5,618 increment cores sampled from 524 open-pollinated families from both trials) (Chen et al., 2014). In 2015, we sampled the second batch of 798 wood disks cut at breast height from another 798 open-pollinated families. A diagram of the development of the plant materials, including the samples used for analyses and references to previous work using the same population, is shown in Supplementary Figure S1.

**Figure 1:**
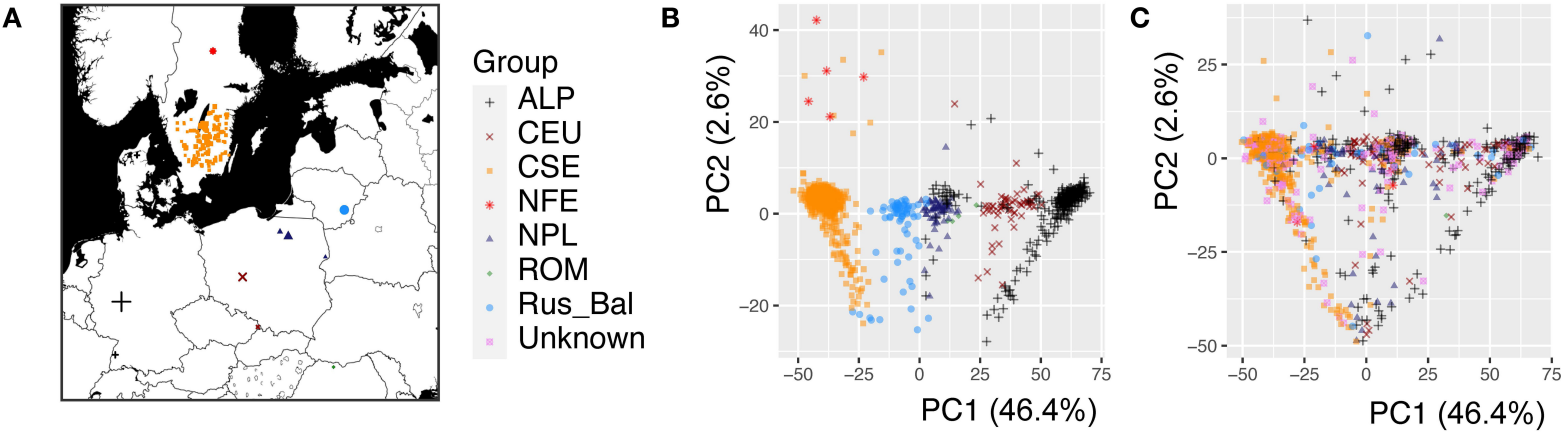
The geographic origin and population structure of the *Picea abies* trees used for the genome-wide association study. **(A)** Geographic distribution of the *P. abies* mother trees (1,080 mother trees genotyped), and the color and shape of the dots represent the genetic cluster indicated in the PCA analysis, **(B)** Population structure of the 1,080 mother trees visualized by the first two principal components (PCs), and **(C)** Population structure of the 1,303 progenies. A total of 1,080 mother trees cluster into seven genetic groups (Chen et al., 2021), but the genetic group is unknown for 223 mother trees. The PCA analysis was performed by combining the parental and progeny datasets. The different genetic clusters were marked (Carpathian including Romania, forest green, ROM; Alpine, black, ALP; Central Europe, dark red, CEU; Northern Poland, midnight blue, NPL; Russia-Baltic, dodgerblue, Rus-Bal; central and southern Sweden, dark orange, CSE; Fennoscandia, red, NFE; Unknown in violet represents unknown progenies).

The mothers of these 1,303 open-pollinated families had been selected from forest stands in Sweden based on their outstanding phenotypic values (e.g., height, diameter, and branch quality) and grafted in two clonal common gardens (Zhou et al., 2019). The geographical origin of the mother trees covers the whole distribution range of Norway spruce, except northern Scandinavia (Figure 1A). We also sampled one increment core from each of the 476 mother trees.

### Phenotyping

In this study, we phenotyped the second batch of 798 woody disks for wood traits using the SilviScan technology (Lundqvist and Evans, 2004) which combines X-ray, microscopy, and image analysis at RISE (https://www.ri.se/en, Stockholm, Sweden). The annual ring width, number of cells, radial and tangential tracheid width, and cell-wall thickness were recorded from microscopy images and image analysis from the pith to the bark for consecutive radial intervals of 25 μm. Wood density (WD) was measured by X-ray absorption at a sampling interval of 25 μm and microfibril angle (MFA) by X-ray diffraction at a sampling interval of 5 mm as previously described (Lundqvist and Evans, 2004). Wood coarseness, number of cells, and wood stiffness expressed as modulus of elasticity (MOE) were predicted based on SilviScan measured traits (Supplementary Methods S1). We combined SilviScan data from the two batches of woods (*n* = 1,303) for our GWAS analysis. The SilviScan data of 476 mother trees were used for candidate SNP validation (Zhou et al., 2019).

#### Juvenile wood (JW), mature wood (MW), and whole core wood (WCW)

**Figure 2:**
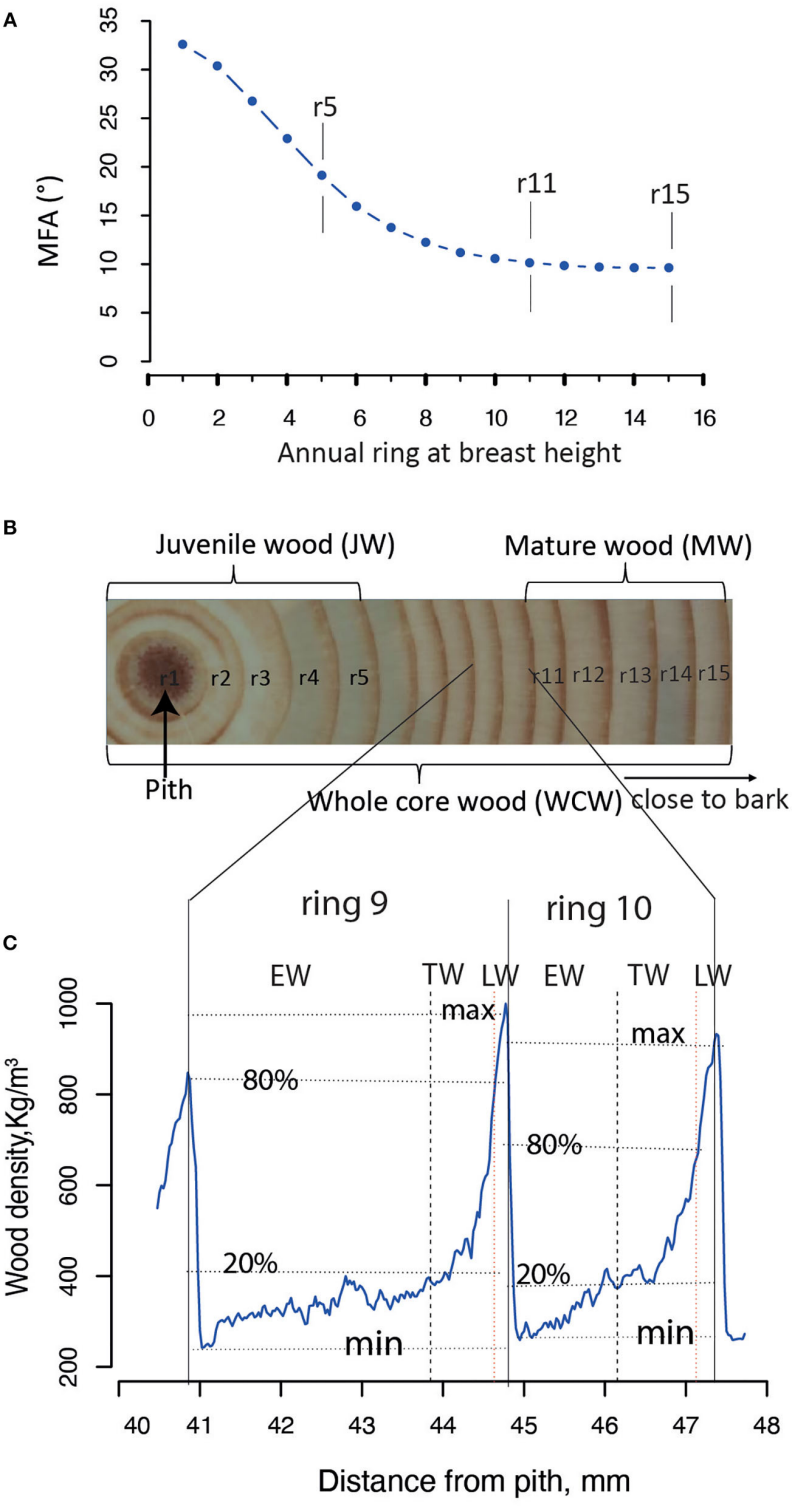
Definition of different wood sections in *Picea abies*. **(A)** The mean microfibril angle (MFA) for each annual ring at the breast height (1.3 m). All the SilviScan samples from Höreda trial were used to estimate the year ring mean value for MFA. **(B)** The juvenile wood (JW) and mature wood (MW) were defined based on a previous study (Hayatgheibi et al., 2018); JW: rings 1–5, MW: rings 11–15. The whole core wood (WCW) was defined as year rings from pith to bark (rings 1–15). **(C)** Each annual ring wood (ARW, e.g., rings 9 and 10) was demarcated into earlywood (EW), transition wood (TW), and latewood (LW), based on a “20–80” wood density threshold definition according to the previously published paper (Lundqvist et al., 2018).

The JW and MW were demarcated according to the age curve of MFA for Norway spruce (Hayatgheibi et al., 2018). It was observed that MFA was high (above 20°) until age five, but decreased after that and stabilized at around age 10 (ca. 10°). The distribution of the number of annual rings per sample for our GWAS population is shown in Supplementary Figure S2. In both datasets (*n* = 798 and *n* = 505), we defined the annual rings 1–5 from pith as the JW and the rings 11–15 from pith as the MW. We also defined the wood from the annual ring 1 to ring 15 as the whole core wood (WCW) (Figures 2A,B). In this way, the phenotypic traits from annual rings of the same age were analyzed for GWAS in the two different batches of trees. A total of 34 traits, obtained from each of the JW, MW, and WCW, are shown in Table 1.

**Table 1:** List of the measured traits, their abbreviations, the number of independent SNPs detected, and the pedigree-based narrow-sense heritability for three wood types (JW/MW/WCW) based on 524 half-sib families.

**Trait (abbreviation)**	**BLINK**	**UV-GEMMA**	**Heritability**
	**JW/MW/WCW**	**JW/MW/WCW**	**JW/MW/WCW**
**Annual ring wood ring width (RW)**	0/0/5	0/0/0	0.23/0.38/0.59
*Earlywood ring width* (*ERW*)	0/0/0	0/1/0	0.18/0.35/0.48
*Transition wood ring width* (*TRW*)	0/0/2	0/0/0	0.25/0.29/0.52
*Latewood ring width* (*LRW*)	0/2/0	0/6/0	0.13/0.26/0.49
**Annual ring wood density (WD)**	0/1/0	0/0/0	0.32/0.58/0.67
*Earlywood density* (*EWD*)	0/6/1	1/0/5	0.25/0.56/0.84
*Transition wood density* (*TWD*)	1/1/0	0/0/0	0.32/0.51/0.64
*Latewood density* (*LWD*)	5/1/0	0/0/0	0.30/0.53/0.67
**Annual ring wood radial tracheid width (RTW)**	0/0/0	0/0/0	0.41/0.43/0.57
*Earlywood radial tracheid width (ERTW)*	0/2/0	0/0/0	0.31/0.42/0.61
*Transition wood density (TWD)*	0/0/0	0/0/0	0.38/0.40/0.57
*Latewood density (LWD)*	0/0/0	0/0/0	0.26/0.26/0.49
**Annual ring wood rangential tracheid width (TTW)**	0/0/0	0/0/0	0.11/0.32/0.37
*Earlywood tangential tracheid width* (*ETTW*)	1/0/1	0/0/1	0.03/0.28/0.38
*Transition wood tangential tracheid width* (*TTTW*)	0/0/0	0/0/0	0.12/0.28/0.34
*Latewood tangential tracheid width* (*LTTW*)	0/0/0	0/0/0	0.11/0.25/0.28
Annual ring wood wall thickness (WT)	0/0/0	0/1/1	0.26/0.51/0.54
***Earlywood wall thickness*** **(*****EWT*****)**	0/2/1	0/0/0	0.18/0.49/0.60
*Transition wood wall thickness* (*TWT*)	0/1/0	0/0/0	0.26/0.39/0.47
*Latewood wall thickness* (*LWT*)	0/0/0	1/0/0	0.25/0.41/0.51
**Annual ring wood coarseness (C)**	0/0/0	0/0/2	0.20/0.33/0.37
*Earlywood coarseness* (*EC*)	0/0/0	0/0/0	0.12/0.37/0.39
*Transition wood coarseness* (*TC*)	0/0/4	0/0/0	0.20/0.25/0.33
*Latewood coarseness* (*LC*)	0/2/0	3/0/0	0.17/0.22/0.29
Annual ring wood number of cells (NC)	0/0/7	0/0/0	0.27/0.38/0.50
*Earlywood number of cells* (*ENC*)	0/0/0	0/1/0	0.18/0.36/0.28
*Transition wood number of cells* (*TNC*)	0/0/1	0/3/0	0.28/0.27/0.36
*Latewood number of cells* (*LNC*)	0/0/1	0/0/0	0.14/0.26/0.14
*Earlywood percentage* (*EP*)	0/0/3	0/0/2	0.24/0.19/0.32
*Transition wood percentage* (*TP*)	0/0/1	0/0/0	0.15/0.09/0.29
*Latewood percentage* (*LP*)	0/0/0	0/0/0	0.06/0.17/0.20
*Early/Latewood percentage* (*EP/LP*)	0/0/0	0/0/0	0.02/0.25/0.26
**Annual ring wood microfibrial angle (MFA)**	1/0/0	0/0/0	0.27/0.13/0.16
**Annual ring wood modulus of elasticity (MOE)**	0/0/1	0/0/0	0.31/0.38/0.43
Total no. of SNPs or average heritability*	8(8)/18(16)/28(23)	5(4)/12(8)/11(9)	0.21/0.34/0.44

* The value is the sum of the total number of SNPs for each statistical method and each type of wood. The SNPs detected for different traits were counted as independent SNPs. The values in parenthesis are the total number of independent SNPs/associations.

JW, juvenile wood; MW, mature wood; WCW, whole core wood. The traits in bold were from the annual ring wood (ARW).

#### Earlywood (EW), transition wood (TW), and latewood (LW)

The annual ring wood (ARW) was divided into EW, TW, and LW based on WD within the ring of each year (Figure 2C). Ring area with WD <20%, between 20 and 80%, and more than 80% of the maximum WD was defined as EW, TW, and LW, respectively (Lundqvist et al., 2018).

### Genotypic data

#### DNA extraction, sequence capture, and SNP calling

Buds and needles were collected from 1,303 progenies and 476 mother trees. Thereafter, total genomic DNA was extracted using the Qiagen Plant DNA extraction protocol with DNA quantification performed using the Qubit^®^ ds DNA Broad Range Assay Kit (Qiagen, Oregon, USA). Exome sequence capture was performed using the 40,018 probes previously designed and evaluated for these materials (Vidalis et al., 2018), and the samples were sequenced to an average depth of 15x in an Illumina HiSeq 2500 platform. The probes were designed to be located inside a coding region. Raw reads based on 2 × 100 bp sequencing mode were mapped to the *P. abies* reference genome v1.0 using BWA-mem (Langmead and Salzberg, 2012). The details of the SNP calling are found in Supplementary Methods S2.

#### Quality control of called SNPs

Several steps of quality control, such as removing indels and sites with call rates < 70%, were performed using VCFtools (Danecek et al., 2011) (Supplementary Methods S3). After these filtering steps, a total of ~300K SNPs with minor allele frequency (MAF) >0.005 were left for population structure analysis. Beagle v4.1 was used to impute the missing genotypes. The mean accuracy of the imputed genotypes was 0.97 at both individual and SNP levels based on a full dataset [ca. 8,000 individuals including the dataset published in Chen et al. (2021)].

### Statistical analysis

#### Adjusting phenotypic values

Tree height and diameter at breast height (DBH) were measured for all the trees (*n* = 12,844) in the Höreda trial. The wood quality traits were only measured for one to a few individuals per family. Since a strong spatial effect was earlier observed in this trial (Chen et al., 2017), multivariate linear mixed models were employed to adjust for the environmental effects (Supplementary Methods S4).

#### Estimation of the variance components

Variance components were estimated by fitting a univariate linear mixed model for the 1,303 unrelated progeny trees


(1) $$\begin{array}{r} y=X\beta +Za+e \end{array}$$

where *y* is the vector of adjusted phenotypic trait, β is the vector of fixed effect including an intercept as the grand mean, and *a* is the vector of random additive effects, following $a\sim \;N\left(0,\;G\sigma_{a}^{2}\right)$. *G* is the genomic-based relationship matrix (GRM) estimated based on the method “VanRaden” (VanRaden, 2008) using AGHmatix package in R (Amadeu et al., 2016). $\sigma_{a}^{2}$ is the additive variance. ***X*** and ***Z*** are the related design matrices of β and *a*, and *e* is the vector of residuals. All analyses were done using ASReml R v4.0.

To compare pedigree-based narrow-sense heritability with SNP-based narrow-sense heritability, we performed a pedigree-based model fitting using SilviScan data from 5,618 increment cores of the 524 half-sib families from the two sites in Horeda and Maltesholm, as described and used in the previous paper (Chen et al., 2014). Equations of estimating the pedigree-based narrow-sense heritability and SNP-based narrow-sense heritability are shown in Supplementary Methods S5.

### Population structure

The population structure of the mother trees has been described for a much larger population in a previous study (Chen et al., 2021). Here, the population structure of the mothers and the progenies was visualized by principal component analysis (PCA) using the *prcomp* function in R v3.6.1.

### SNP-trait association

We performed GWAS by three methods. The first method was BLINK which conducts two fixed-effect models and one filtering process (Huang et al., 2019). The details of the method are given in Supplementary Methods S6. The second method was univariate GWAS (UV-GEMMA), which was performed using genome-wide efficient mixed-model analysis (GEMMA). The third method was multivariate GWAS (MV-GEMMA), which was also performed using GEMMA (Zhou and Stephens, 2014). After filtering locus with MAF < 0.03, 131,131 SNPs were used in GWAS. Univariate GWAS was run for the 34 traits from each developmental stage of wood formation (Table 1). The UV-GEMMA from GEMMA was as follows:


(2) $$\begin{array}{r} y=W\alpha +X\beta +Zu+\epsilon \end{array}$$

where *y* is the vector of adjusted phenotypic values, α is a vector of corresponding fixed effects including the intercept and the principal components/admixture Q matrix if it is fitted, β is a vector of the marker effects, *u* is a vector of the polygenetic additive effects, and ϵ is a vector of residuals. ***W*** , ***X*** , and ***Z*** are the related design matrices. Population structure was considered if the genomic inflation factor (IF), which expresses the deviation of the distribution of the observed test statistic compared to the distribution of the expected test statistic, is not within 1 ± 0.05 (Yang et al., 2011). However, because the values of IF were all within 1 ± 0.05 (Supplementary Figure S3), the population structure in Equation (2) was excluded in the later analysis. The details of the MV-GEMMA model are provided in Supplementary Methods S7. The percentage of variation explained (PVE) by each SNP was estimated using the formula in Shim et al. (2015). The cumulative PVE of all candidate SNPs for each trait was estimated using a genomic prediction model within all candidate SNPs for each trait as fixed effects (Supplementary Methods S8).

### Trait selection for GEMMA multivariate GWAS

Multivariate sets of traits were created based on phenotypic or genetic correlations among the phenotypes, and structural and functional relationships of the traits (Chen et al., 2014, 2016). A moderate phenotypic and/or genetic correlation could be important for fitting multivariate GWAS (Stephens, 2013). Therefore, to obtain interesting multivariate sets, we estimated pairwise phenotypic correlations. Finally, 21 multivariate sets (Table 2) for each type of wood (JW, MW, and WCW) were selected as traits for multivariate GWAS. Based on phenotypic correlation and different selection criteria, we separated these multivariate sets into three types: (a) multivariate set based on the same trait along with seasonal change, such as ring width of earlywood (ERW), transition wood (TRW), latewood (LRW), and annual wood ring wood (ARW), named as RW(ARW_EW_TW_LW); (b) multivariate set based on the phenotypic correlation among traits from ARW, e.g., ring width, WD, coarseness, and number of cells, named as ARW(RW_WD_C_NC); and (c) multivariate set based on the mathematical relationship among traits from ARW, such as modulus of elasticity = WD × MFA, called ARW(MOE_WD_MFA).

**Table 2:** List of 21 multivariate sets used for multivariate GEMMA (MV-GEMMA), their abbreviations, and the number of independent SNPs detected using MV-GEMMA.

**Phenotypic trait**		**No. of SNPs**
**1) Multivariate set based on the seasonal variation**	**Abbreviation**	**JW/MW/WCW**
**Ring width** (*ARW, EW, TW, LW*)	RW(ARW_EW_TW_LW)	0/0/0
**Wood density** (*ARW, EW, TW, LW*)	WD(ARW_EW_TW_LW)	1/1/5
**Radial tracheid width** (*ARW, EW, TW, LW*)	RTW(ARW*_*EW_TW_LW)	0/0/0
**Tangential tracheid width** (*ARW, EW, TW, LW*)	TTW(ARW_EW_TW_LW)	0/0/2
**Wall thickness** (*ARW, EW, TW, LW*)	WT(ARW_EW_TW_LW)	0/0/2
**Coarseness** (*ARW, EW, TW, LW*)	C(ARW_EW_TW_LW)	0/0/0
**Number of cells** (*ARW, EW, TW, LW*)	NC(ARW_EW_TW_LW)	0/0/5
**Percentage** (*AEW, TW, LW, EP/LP*)	P(EW_TW_LW_EP/LP)	0/0/0
**2) Multivariate set based on the phenotypic correlation**		
**ARW** (*Ring width, wood density, coarseness, number of cells*)	ARW(RW_WD_C_NC)	0/0/0
**EW** (*Ring width*, wood *density, coarseness, number of cells*)	EW(RW_WD_C_NC)	0/1/1
**TW** (*Ring width*, wood *density, coarseness, number of cells*)	TW(RW_WD_C_NC)	0/1/2
**LW** (*Ring width*, wood *density, coarseness, number of cells*)	LW(RW_WD_C_NC)	0/0/0
**3) Multivariate set based on the predicted trait**		
**ARW** (*Coarseness^#^, wood density, radial tracheid width, tangential tracheid width*)	ARW(C_WD_RTW_TTW)	0/2/1
**EW** (Coarseness*^#^*, wood density, radial tracheid width, tangential tracheid width)	EW(C_WD_RTW_TTW)	0/3/3
**TW** (Coarseness*^#^*, wood density, radial tracheid width, tangential tracheid width)	TW(C_WD_RTW_TTW)	0/0/0
**LW** (Coarseness*^#^*, wood density, radial tracheid width, tangential tracheid width)	LW(C_WD_RTW_TTW)	0/0/0
**ARW** (*Number of cells^#^, ring width, radial tracheid width*)	ARW(NC_W_RTW)	1/0/0
**EW** (*Number of cells^#^, ring width, radial tracheid width*)	EW(NC_W_RTW)	0/1/0
**TW** (*Number of cells^#^, ring width, radial tracheid width*)	TW(NC_W_RTW)	0/0/1
**LW** (*Number of cells^#^, ring width, radial tracheid width*)	LW(NC_W_RTW)	0/0/0
**ARW** (*Modulus of elasticity ^#^, wood density, mirofibril angle*)	ARW(MOE_WD_MFA)	1/0/0
Total no. of SNPs*		3(3)/9(5)/22(11)

EW, earlywood; TW, transition wood; LW, latewood; ARW, annual ring wood; JW, juvenile wood; MW, mature wood; WCW, whole core wood.

^#^Presents that the trait was predicted by predictor traits. For example, MOE = WD × MFA, in which MOE, WD, and MFA represent the modulus of elasticity, wood density, and microfibril angle, respectively. Number of cells represents ring width divided by radial tracheid width.

*Represents that the value is the sum of the total number of SNPs for traits from each of three wood types (JW, MW, and WCW) and also the same SNPs here detected by different traits were counted as different SNPs, and the values in the parenthesis are the total number of independent associations (SNPs) for each wood type.

### Spatial and temporal expression pattern of the associated candidate genes

Data for the spatial and seasonal expression patterns of the identified candidate genes were retrieved from previously published RNA-seq analyses in Norway spruce (Jokipii-Lukkari et al., 2017, 2018), downloaded from plantgenie.org. The heatmaps are visualized by the pheatmap function in the pheatmap R package (Kolde and Kolde, 2015).

### Validation of the GWAS signals using parental population

Finally, we performed validation tests using a randomly selected part (*n* = 476) of the 1,303 mother trees. A mixed linear model (MLM) with a genomic-based relationship matrix (GRM) was used to test whether an SNP was associated with a trait (Supplementary Methods S9) (Zan and Carlborg, 2018).

### Variant annotation for significant associations

Significantly associated SNPs at a 5% false discovery rate (FDR) were annotated using SnpEff 4.3 with default parameters (Cingolani et al., 2012). The general transfer format (GTF) from Ensembl was utilized to build the *P. abies* SnpEff database. To summarize the results, significant SNPs within a gene model were merged and counted as a single significant locus. To assess the variant effect of the associated SNPs, annotation of the putative genes, genome ontology (GO), and their associated *Arabidopsis* and *Populus* orthologs were performed using the *P. abies* v1.0 genome on ConGenIE database (Sundell et al., 2015). All genes within a contig harboring the associated SNPs were extracted from ConGenIE. Due to the repetitive nature of conifer genomes (Niu et al., 2022), the candidate genes located in the same contig harboring an associated SNP and annotated with a similar biological function were also extracted in this study.

## Results

In this study, 1,303 Norway spruce progenies were analyzed for growth and wood properties to investigate the effect of tree age and seasonal cycle on the underlying genetic architecture. A large amount of variation in these properties was expected due to the rather wide geographic distribution of the mother trees (Figure 1A). In a previous study (Chen et al., 2021), the mother trees of these progenies were clustered into seven genetic groups (Figure 1B). The clustering of the progenies that were obtained by open pollination was slightly different from that of the mother trees (Figure 1C). These results suggest that population structure is very important in F1 generation/progeny and should be fitted in Norway spruce traditional genetic analysis, genomic selection, and GWAS.

### Estimates of heritabilities for tree growth and wood properties

**Figure 3:**
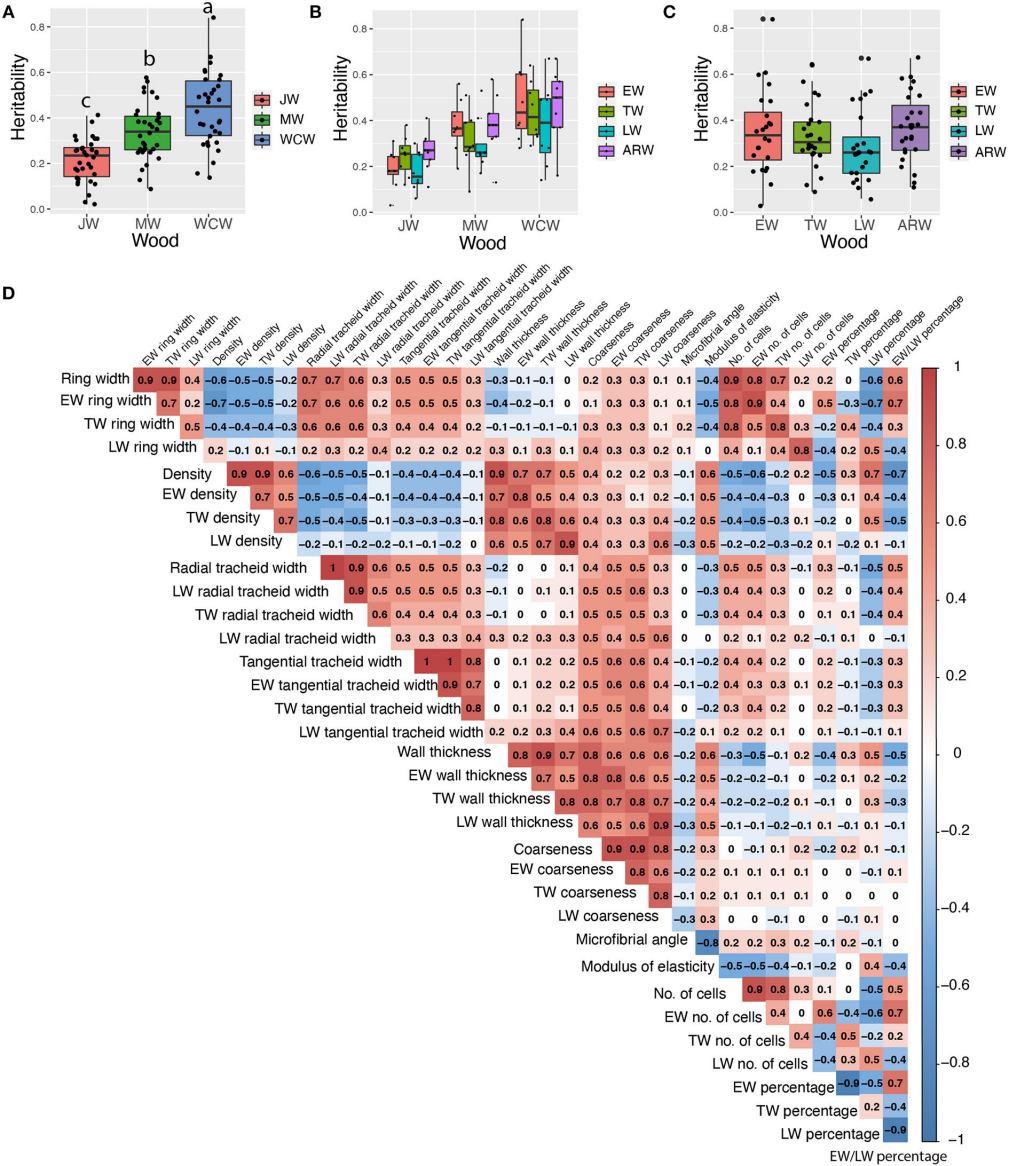
Estimated narrow-sense heritabilities of growth and wood quality traits and pairwise Pearson phenotypic correlations among all wood traits from the whole core wood (WCW). **(A)** Boxplots of pedigree-based narrow-sense heritabilities of 34 traits from juvenile (JW), mature (MW), and WCW using 505 half-sib families. **(B)** Boxplots of pedigree-based heritabilities of 34 traits from earlywood (EW), transition wood (TW), latewood (LW), and annual ring wood (ARW) using 505 half-sib families. **(C)** Boxplots of pedigree-based heritabilities for growth and wood quality traits produced at different seasons in JW, MW, and WCW using 505 half-sib families. **(D)** Pairwise Pearson phenotypic correlations among all wood traits from the whole core wood (WCW). The color spectrum indicates highly positive (red) to highly negative (blue) correlations, and the number indicates the correlation values. The blank indicates a lack of significant correlation (*P* > 0.01). In **(A)**, the different letters between mean heritabilities in JW, MW, and WCW represent significant differences (*P* > 0.01).

We estimated both pedigree- and SNP-based narrow-sense heritabilities of 34 traits for each developmental stage of wood formation (Supplementary Table S1). Overall, SNP-based heritabilities were much lower than the pedigree-based heritabilities, except for tangential tracheid width (TTW) from JW. As we expected, SNP-based heritability (based on a single tree per family) had a larger standard error than that of pedigree-based heritability (based on 12 trees per family). Thus, we focused only on the results from the pedigree-based heritability in the following sections (Table 1). On average, traits from JW had significantly lower heritability (0.21 ± 0.02) than those obtained from the MW (0.34 ± 0.02) and WCW (0.44 ± 0.03) (Figure 3A). Traits from EW, TW, and ARW had higher average heritability than those obtained from LW in JW, MW, and WCW (Figure 3B) and in all three kinds of wood (Figure 3C), but the difference was non-significant. In general, the traits related to wood density (WD) showed higher heritabilities than most other traits (Table 1). Taken together, the range of heritabilities suggests that the environment shapes the wood traits more in the JW than in the MW or WCW, and more in the LW than in the EW, TW, or ARW.

### Phenotypic correlation between traits

Phenotypic correlations were analyzed between all the measured traits in the whole wood (whole core wood, WCW) (Figure 3D) by Pearson pairwise correlation analysis. As expected, growth-related traits, such as ring width, number of cells, and tracheid number, correlated positively with each other. Wood density (WD) also showed an expected positive correlation with cell wall thickness and a negative correlation with the ring width. Similar correlations were observed in JW (Supplementary Figure S4) and MW (Supplementary Figure S5). The seasonal effect was represented by the earlywood/latewood ratio that correlated positively with the ring width but negatively with wood density and cell wall thickness (Figure 3D).

Genetic association analyses can be done for either single traits or simultaneously for multiple traits. As several of the traits analyzed here had moderate to high correlations with each other (Figure 3D), multivariate sets of traits were identified that could be utilized in our genetic studies. For each developmental stage (JW, MW, and WCW), 21 sets of traits (Table 2) were identified based on moderate to high phenotypic correlations (Figure 3D). In group 1, eight multivariate sets were selected based on a high positive pairwise correlation of traits within EW, TW, and LW. In group 2, four multivariate sets were selected based on a moderate phenotypic correlation between traits within the same seasonal and developmental stage of the wood within the rings (for instance, all traits from EW of JW). In group 3, each set was selected from the SilviScan data for a predicted trait and its predictor traits within the same part of the wood (Table 2). These predicted traits, including coarseness, number of cells, and modulus of elasticity (MOE), also showed moderate to high phenotypic correlations with the predictor traits (Figure 3D).

### Genome-wide association between growth and wood traits

We conducted GWAS using univariate BLINK and GEMMA (UV-GEMMA) for 34 traits and multivariate GEMMA (MV-GEMMA) for 21 multivariate sets from each of JW, MW, and WCW. We found that all the associated SNPs within a contig were in strong LD (*r*^2^ > 0.2, Supplementary Table S2). Thus, we considered the SNPs that occurred within the same genomic contig as one independent association. We also considered the SNPs simultaneously detected by two or more traits as one independent association. In total, we identified 74 independent SNPs that were significantly associated with 63 of the altogether 102 traits after multiple testing corrections with the FDR-adjusted *p* < 0.05 (Supplementary Tables S2, S3, and Table 1). These SNPs were located within 74 gene models (Supplementary Table S4). In addition, there were another 11 gene models located nearby these SNPs (±20 kb) within the same contigs, amounting to a total of 85 gene models derived from the GWAS analysis (Supplementary Table S4). A subset of the important genes based on possible biological meaning is presented in Table 3. The proportion of phenotypic variance explained (PVE) by each candidate SNP ranged from ca. 0 to 4.23%. Cumulative PVEs of all candidate SNPs for each trait ranged from 0 to 9.8% (Supplementary Table S5). We also tested the associated SNPs in a subset of the mother population (*n* = 476) and found that nine independent SNPs were significantly associated with the respective traits also in this population (*P* < 0.05) (Supplementary Table S6).

**Table 3:** A selection of genes with significant SNPs associated with different traits in juvenile, mature, and whole core wood in the three different types of GWAS analyses.

**Gene model**	**Method**	**Wood type**	**Abbreviation of trait**	***P*** **-value**	**Description**
MA_10117117g0010*	UV_GEMMA	WCW	EWD	3.77E-07	Mitogen-activated kinase kinase kinase NPK1-like
MA_10277463g0010*	BLINK	JW	TWD	9.66E-08	3-phosphoinositide-dependent kinase-1
MA_10428113g0010	BLINK	MW	EWD	5.97E-07	Cysteine synthase-like
MA_10428864g0010*	MV-GEMMA	WCW	NC(ARW_EW_TW_LW)	2.21E-06	Anaphase-promoting complex subunit 1
MA_106297g0010*	UV_GEMMA	WCW	EWD	3.77E-07	Mitogen-activated kinase kinase kinase NPK1-like
MA_12842g0010/20/30*^#^	UV_GEMMA^#^	WCW^#^	EWD^#^	3.77E-07	Mitogen-activated kinase kinase kinase NPK1-like
MA_436199g0010*	MV-GEMMA	WCW	LW(C_WD_RTW_TTW)	2.02E-08	Chloroplast beta-amylase (AtBAM3)
MA_879701g0010*	MV-GEMMA	JW	WD(ARW_EW_TW_LW)	6.45E-07	Unknown
MA_14038g0010	BLINK	JW	LWD	5.37E-07	GATA transcription factor 12-like
MA_464588g0010	BLINK	WCW	RW	5.77E-08	BIG GRAIN 1-like A
MA_8964699g0010*	BLINK	MW	LC	6.07E-09	MOTHER of FT and TFL1-like isoform X2
MA_95898g0010	BLINK	MW	LC	9.81E-08	No apical meristem
MA_183130g0010*	MV_GEMMA	WCW	TW(RW_WD_C_NC)	5.21E-07	Cellulose synthase A7 (AtCesA7)
MA_183130g0020	MV_GEMMA	WCW	TW(RW_WD_C_NC)	5.21E-07	Cellulose synthase A4 (AtCesA4)
MA_29357g0010	UV_GEMMA	WCW	EW(WD_RTW_TTW_C), WD(ARW_EW_TW_LW), WT(ARW_EW_TW_LW)	9.95E-08	Mitogen-activated kinase kinase kinase NPK1-like
MA_5468g0010	BLINK	WCW	EP, TTW(ARW_EW_TW_LW)	1.41E-10	Nascent polypeptide-associated complex subunit alpha 1
MA_64117g0010	BLINK	WCW	RW	3.42E-07	Leucine-rich repeat receptor-like serine threonine- kinase BAM1
MA_77420g0010	MV_GEMMA	JW	ARW(NC_RW_RTW)	1.39E-07	Ethylene-responsive transcription factor ESR2-like

Gene model is annotated using v1.0 of the Picea abies genome. JW, juvenile wood; MW, mature wood; WCW, whole core wood. *Represents that the gene with validated SNPs under p < 0.05 in the mother population. ^#^Represents that there are three genes: MA_12842g0010, MA_12842g0020, and MA_12842g0030. The three genes were detected by all three methods, and also in JW, MW, and WCW, and many other traits, as shown in Supplementary Table S6. Please refer to Tables 1, 2 for full name of the abbreviation of traits

### The effect of tree age on the genetic architecture of growth and wood properties

**Figure 4:**
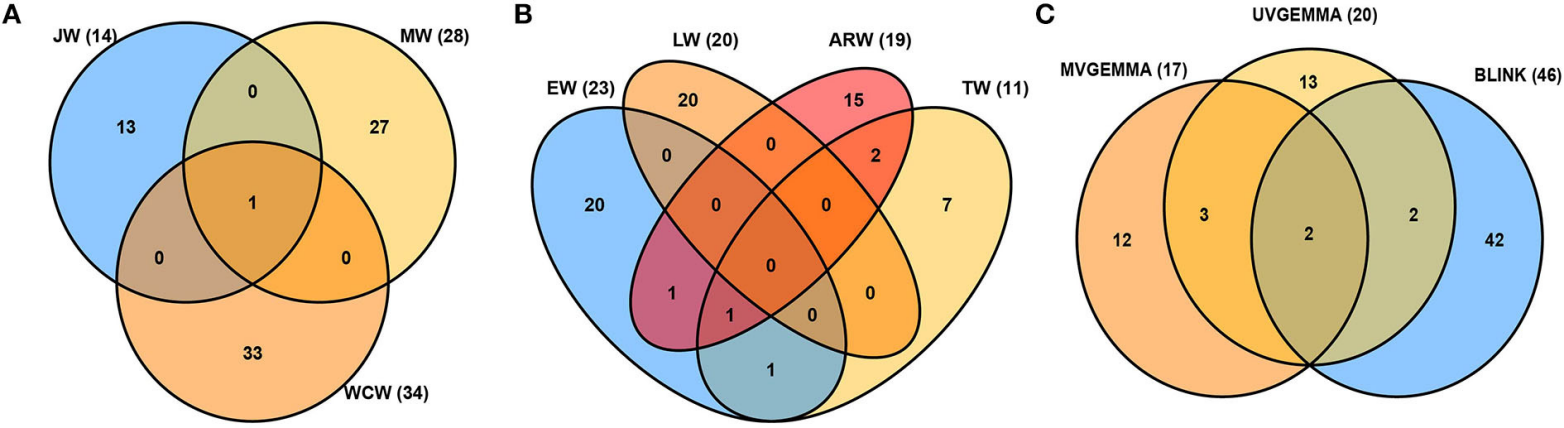
Venn diagrams of associated single-nucleotide polymorphisms (SNPs) detected for all traits. **(A)** Juvenile wood (JW), mature wood (MW), and whole core wood (WCW). **(B)** Earlywood (EW), transition wood (TW), latewood (LW), and annual ring wood (ARW). **(C)** Comparison of the univariate GEMMA (UV-GEMMA), multivariate GEMMA (MV-GEMMA), and univariate BLINK (BLINK) GWAS methods. The value inside the parenthesis is the number of associated SNPs.

Tree aging is concomitant with the transition from JW to MW formation. We found in the current population that JW was formed typically until the annual ring 5 at breast height and MW after the annual ring 10 (Figure 2). To estimate the effect of these developmental changes on the genetic architecture of growth and wood properties, we surveyed the SNPs identified by GWAS for traits in JW, MW, and WCW (Figure 4A). Among them, only one SNP from contig MA_12842 was shared by the different age classes. These results suggest that the genetic architecture of the wood traits is largely dependent on the age of the trees.

As an example of an association specific to JW, an SNP in the gene model MA_77420g0010, encoding a putative member of the ethylene response factor family, was associated with the multi-traits composed of the number of cells, ring width, and tangential ring width (Table 3). The homologous *Arabidopsis thaliana* gene AT1G24590 controls organogenesis and is also linked to provasculature development (Glowa et al., 2021). An SNP in another gene model MA_14038g0010, encoding a member of the GATA transcription factor family, was associated with latewood density in JW (Table 3).

**Figure 5:**
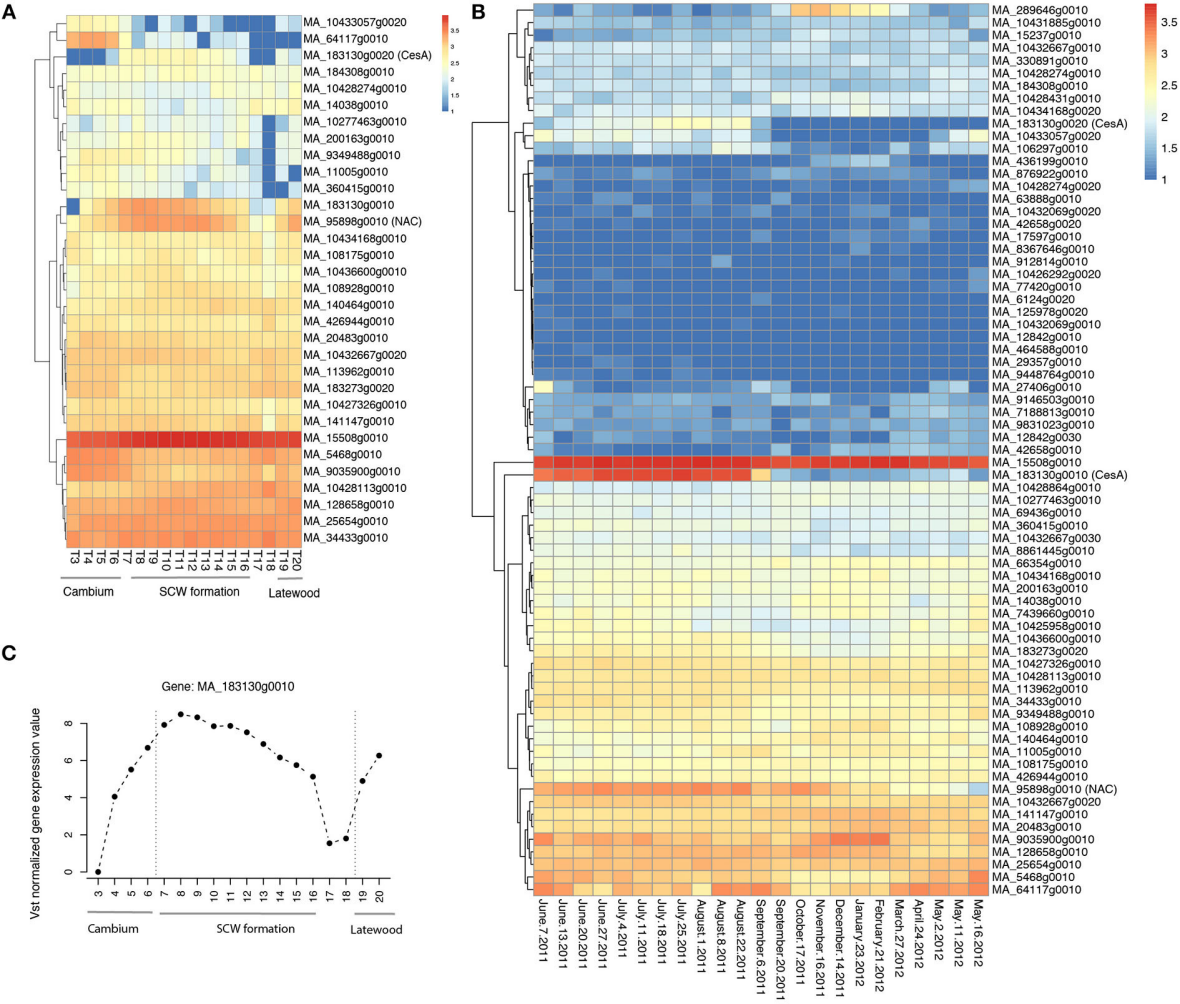
Spatial and temporal expression pattern of the identified candidate genes in Norway spruce. **(A)** Heatmap of RNA-seq data from the different wood developmental zones of Norway spruce stem (tree 1; Jokipii-Lukkari et al., 2017); **(B)** Heatmap of RNA-seq data in a whole seasonal cycle for the xylem of Norway spruce; **(C)** The expression of the candidate gene MA_183130g0010 in Norway spruce (tree 1; Jokipii-Lukkari et al., 2017). Vst means variance-stabilizing transformation. The data used in **(A–C)** can be downloaded from the website (https://plantgenie.org). SCW is the secondary cell wall.

In MW, an SNP in MA_95898g0010 was associated with LW coarseness (LC). MA_95898g0010 is a member of the NAC transcription factor family. The expression of MA_95898g0010 varies according to different developmental zones (Figure 5A) and the season (Figure 5B) and is strictly coregulated with secondary cell wall CesAs in Norway spruce wood (Jokipii-Lukkari et al., 2017). Another interesting SNP was located in the gene model MA_8964699g0010, which, similar to the SNP in MA_95898g0010, was associated with the LW coarseness in MW. MA_8964699g0010 is annotated in Norway spruce as MOTHER OF FT AND TFL1-like and validated in the mother population (Table 3).

When considering the whole core wood (WCW), associations were found between ring width and SNPs in two gene models that on the basis of the functional characterization of the *Arabidopsis thaliana* (hereafter “Arabidopsis”) homologs putatively promote growth or biomass production. MA_64117g0010 encodes a homolog of Arabidopsis BARELY ANY MERISTEM 1 (BAM1), which is a CLAVATA1-related receptor kinase-like protein required for formative cell divisions and xylem patterning in the root (Crook et al., 2020; Fan et al., 2021). MA_464588g0010, on the other hand, encodes a homolog of rice BIG GRAIN 1, which stimulated biomass accumulation when overexpressed in rice (Liu et al., 2015). These two genes could possibly act to control cell divisions and biomass production from the vascular cambium, which is supported for MA_64117g0010 by its specific expression pattern in the vascular cambium of Norway spruce stem (Figure 5A; see also the Norwood data in https://plantgenie.org; Jokipii-Lukkari et al., 2017).

### The effect of the annual season on the genetic architecture of growth and wood properties

The seasonality of cambial growth is manifested by the transition from EW to LW formation along with the shortening of the day length. GWAS detected a total of 23, 11, 20, and 19 SNPs associated with the traits of EW, TW, LW, and ARW, respectively (Figure 4B). There were no common SNPs between EW and LW, showing contrasting genetic architecture of growth and wood properties not only in response to tree age but also to the seasonal changes.

In EW, GWAS detected two candidate genes (MA_10117117g0010 and MA_29357g0010, annotated as mitogen-activated protein kinase kinase kinase, MAP3K) that were associated with earlywood density and also validated in the mother population (Table 3 and Supplementary Table S6). Three other MAP3Ks (MA_12842g0010, MA_12842g0020, and MA_12842g0030) were also identified in the GWAS analysis. They were associated with several traits, including ring width and WD, and were also validated in the mother population (Table 3 and Supplementary Table S6). All these MAP3K gene models correspond to the Arabidopsis gene AT5G55090 which has been linked to drought resistance (Pieczynski et al., 2018). Increased expression of MA_12842g0030 was observed in early spring and July throughout the season in the woody tissues (Figure 5B), as well as in buds between the early bud stage and late bud stage, of Norway spruce (Chen et al., 2021).

In TW, it was interesting to identify SNPs in a secondary cell wall cellulose synthase (CesA) gene model MA_183130g0010 that associated with a multivariate set of traits (ring width, WD, coarseness, and number of cells) (Table 3; Supplementary Table S7). Also, enhanced expression of MA_183130g0010 was found during the formation of the secondary cell wall (Figure 5A) in the growing season and less in autumn and winter seasons (Figures 5B,C; Jokipii-Lukkari et al., 2018), which together with the GWAS data suggests that transition from earlywood to latewood is associated with increased activity of cellulose synthesis mediated by changes in the expression of these CesAs.

In LW, SNPs were identified in the earlier mentioned *MOTHER OF FT AND TFL1-like* gene (MA_8964699g0010) in association with LW coarseness in MW (Table 3; Supplementary Table S7). Interestingly, this gene belongs to the same gene family as the TERMINAL FLOWER genes TFL1 and TFL2 that have been linked earlier to seasonality in Norway spruce (Klintenäs et al., 2012; Karlgren et al., 2013). A latewood multi-trait LW(C_WD_RTW_TTW) was associated with an SNP in a β-amylase (MA_436199g0010 in Table 3; Supplementary Table S7), which is homologous to an Arabidopsis BAM3 with a proposed function in cold response (Monroe et al., 2014).

### Comparison of results detected by three GWAS methods

**Figure 6:**
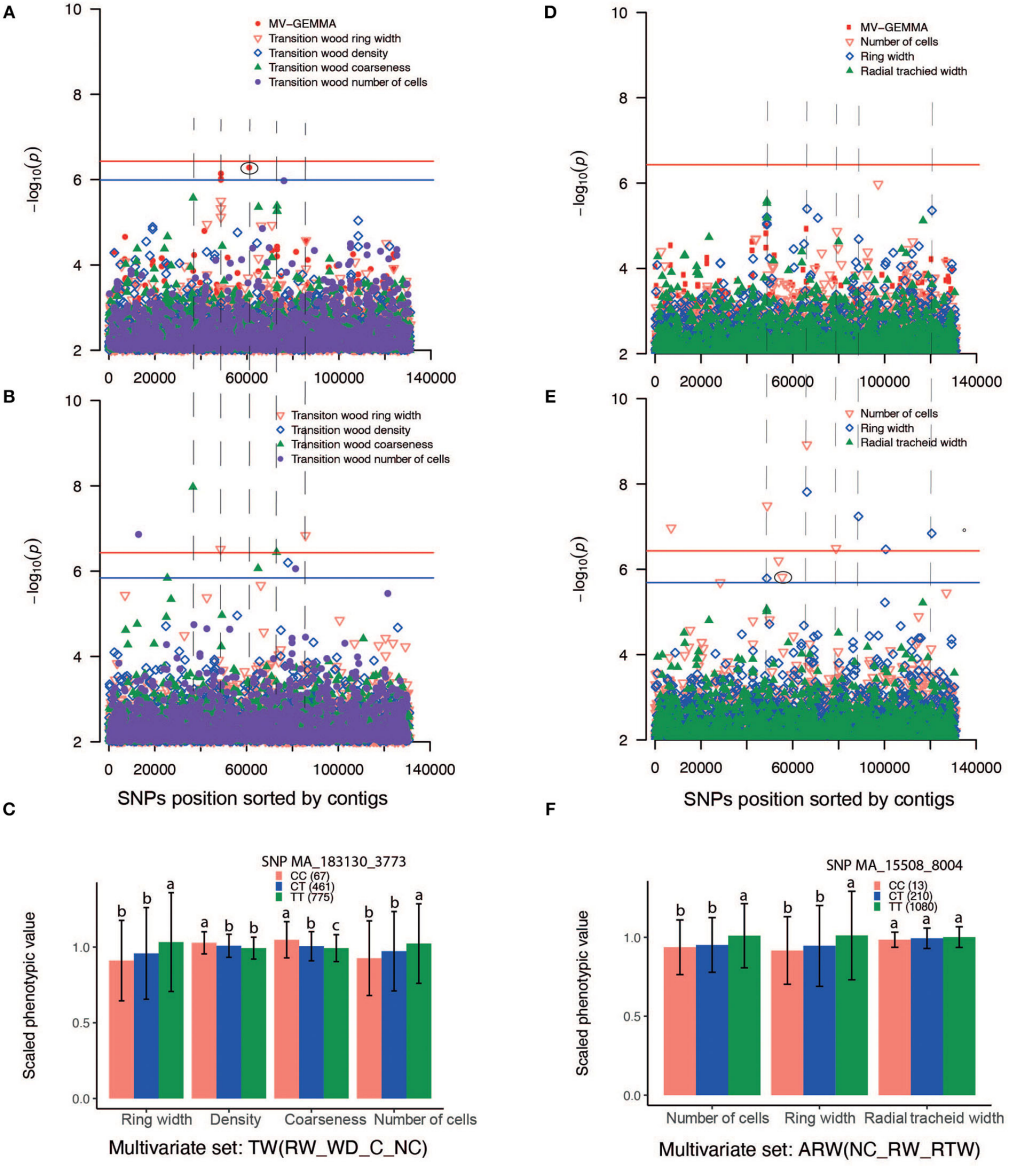
Manhattan plots comparing GEMMA univariate (UV-GEMMA), multivariate (MV-GEMMA), and BLINK GWAS for wood traits measured in *Picea abies*. *P*-values are converted to –log10 (*P*-value). Single-nucleotide polymorphisms (SNPs) above the red lines passed the Bonferroni correction test (*P* < 3.7 × 10^−7^). SNPs above the blue line passed false discovery rate (FDR) (*P* < 0.05) for the multivariate set in **(A)**, transition wood of coarseness in **(B)**, and the number of cells in **(E)** (blue line was only shown for the interesting traits and there is SNP passing FDR *P* < 0.05). Only SNPs with *P* < 1 × 10^−2^ are plotted. **(A)** Manhattan plot based on multivariate (MV-GEMMA) and univariate (UV-GEMMA) analysis of four traits in the transition wood [ring width, wood density, coarseness, and the number of cells, labeled as TW(RW_WD_C_NC)]. **(B)** Manhattan plot based on univariate model BLINK only for the same four traits in the transition wood. **(C)** Allelic effects of SNP MA_183130_3773 on the four traits in the transition wood. CC, CT, and TT are genotypes of the associated SNP. The number in parenthesis is the number of individuals for each of genotypes CC, CT, or TT. **(D)** Manhattan plot based on multivariate (MV-GEMMA) and univariate (UV-GEMMA) analysis of three traits in the annual ring wood [number of cells, ring width, and radial tracheid width, labeled as ARW(NC_RW_RTW)]. **(E)** Manhattan plot based on univariate model BLINK only for the same three traits in the annual ring wood. **(F)** The allelic effects of SNP MA_15508_8004 on the three traits of the annual ring wood. SNPs depicted in **(C,F)** are eclipsed in the corresponding Manhattan plot, and the error bar represents ±standard deviation for phenotypic values. Different letters represent a significant difference (*P* < 0.05) between the mean values of different genotypes. Phenotypic values are scaled by their mean value. The dashed lines linking **(A,B)**, and linking **(D,E)** are drawn to identify if MV-GEMMA or BLINK increases the power in the same contig as UV-GEMMA.

The GWAS method BLINK detected higher number (46) of independent SNPs as compared to UV-GEMMA (20) and MV-GEMMA (17) (Figure 4C). To facilitate the presentation of characters of association with the three statistical methods, we divided the association results into the following three categories. First, adjusted *p-*values of a few SNPs in the MV-GEMMA and BLINK models became significant (i.e., FDR-adjusted *p*-value of the few SNPs became < 0.05) compared with being non-significant in the univariate UV-GEMMA model (e.g., Figures 6A–C). For example, MV-GEMMA increased the significance level to detect SNP MA_183130_3773 (Figure 6A, dot with blue circle), but with a different contig position than UV-GEMMA and BLINK (Figure 6B). Second, adjusted *p-*values of a few SNPs became significant only in BLINK when compared with UV-GEMMA and MV-GEMMA (e.g., Figures 6D–F). Third, the number of associated and independent SNPs in all three methods were the same (e.g., Supplementary Figures S6A–C). QQ plots (Supplementary Figures S7, S8) matching the Manhattan plots in Figure 6 showed a clear improvement in detecting the number of significant SNPs using MV-GEMMA and/or BLINK compared with the UV-GEMMA. QQ plots in Supplementary Figure S9 matching the Manhattan plots for all methods in Supplementary Figure S6 were similar due to an equivalent ability to detect the same number of independent SNPs among the three methods (Supplementary Figure S6).

## Discussion

### Different core genes were involved in the different wood developmental stages

In the present study, we found that the genetic architecture differed significantly between the two developmental stages of juvenile and mature wood formation, as they only shared a single SNP in the GWAS analysis (Supplementary Table S3). Several transcription factors, such as annotated as homologs of AtCesA4, AtCesA7, and AtESR2-like, were among the gene models carrying the SNPs identified by GWAS in the JW or the MW (Table 3). Since the transition from juvenile to mature wood formation dramatically influences the growth and wood properties (Zobel and Jett, 1995), these transcription factors provide interesting tools for future breeding approaches. For instance, improved wood quality is one of the main breeding targets in conifers. For such approaches, it is beneficial that juvenile and mature wood have distinct genetic architectures, and that targeted breeding of juvenile wood does not necessarily affect the properties of the mature wood.

Similar to the tree developmental stage (age), different seasons also influenced the genetic architecture of growth and wood properties in unique ways, as no SNPs were shared between the earlywood and latewood in the GWAS (Figure 4B). Five candidate genes, annotated as mitogen-activated protein kinase kinase kinase (MAP3K) and putatively involved in drought resistance (Pieczynski et al., 2018), were detected only in EW, indicating that abiotic stress tolerance shapes wood formation in spring. Interestingly, MAP3K was associated with spring budburst, frost damage, and stem diameter in an earlier study on Norway spruce (Chen et al., 2021). Specific association of earlywood with SNPs of another gene, the cysteine synthase (MA_10428113g0010) (Table 3), was also reported in an earlier study in white spruce (Beaulieu et al., 2011). The latter parts of the season seemed to be involved in the changes associated with the synthesis of cellulose. A secondary cell wall cellulose synthase (CesA) was identified as a candidate gene in the transition wood, and a NAC transcription factor family MA_95898g0010 which is strictly coregulated in Norway spruce with secondary cell wall CesAs (Jokipii-Lukkari et al., 2018) in the mature wood. These results suggest that secondary cell wall deposition that occurs along with the shortening of the day length might involve unique variants of CesAs and their regulation by the NAC transcription factor MA_95898g0010. Latewood properties were also linked to variation in a β-amylase gene (MA_436199g0010), which is homologous to Arabidopsis cold-induced BAM3. Therefore, our work provides substantial evidence for the seasonal control of wood properties by abiotic factors, such as drought in the spring and cold in the late season.

### More putative associations detected by multi-locus BLINK

Many empirical and simulation studies have shown that multi-locus GWAS methodologies, such as FASTmrEMMA (Wen et al., 2017), FarmCPU (Liu et al., 2016), and BLINK (Huang et al., 2019), have more power than single-locus methods, such as standard univariate GEMMA (Xu et al., 2018; Zhang et al., 2019). Also, the multivariate model (e.g., MV-GEMMA) has more power than the standard univariate models (e.g., UV-GEMMA) (Korte et al., 2012; Zhou and Stephens, 2014). Even though the power and Type I errors of the single-locus and multi-locus GWAS methods were not estimated in this study, we did detect more SNPs using the multi-locus and multivariate models. We found that more SNPs were detected by BLINK than by UV-GEMMA and MV-GEMMA. BLINK theoretically selects a set of pseudo-QTNs that are not in LD with each other as covariates, thus only the independent SNPs would be detected in GWAS (Huang et al., 2019). Theoretically, the multivariate model has more power than these standard univariate models because missing data in one of the phenotypic traits could be complemented by other phenotypes based on population correlation (Porter and O'Reilly, 2017). In the present study, however, there is only a slight difference between the number of associations for the two methods. The fact that there was no missing data for the phenotypic traits could be the main reason.

### Effect of population size and heritability on GWAS

One of the main issues for GWAS is the validation of detected QTLs. Most of the GWAS in crops and tree species used ~500 individuals/genotypes (Huang et al., 2010; Hall et al., 2016; Fang et al., 2017; Chhetri et al., 2019; Chen et al., 2021). Few, if any, of the SNPs were validated or repeated in similar or even smaller population size (McKown et al., 2014a,b; Chhetri et al., 2019). For example, Elfstrand et al. (2020), performing GWAS for disease resistance traits in Norway spruce using 466 genotypes, did not find any overlapped SNPs with another previous study using 66 genotypes/clones (Mukrimin et al., 2018). We also did not observe the same QTLs detected in two previous studies of Norway spruce using a smaller population (517 trees) (Baison et al., 2019, 2020). In this study, we performed GWAS analysis in a parental population (476 trees) and identified nine SNPs with *p* < 0.05 that overlapped with the associated SNPs in the GWAS of their progenies (Table 3). We find that three candidate genes annotated as MAP3K for ring width (i.e., diameter) and located in the same contig MA_12842 were observed in a previous study using a larger population (~5,000 trees) (Chen et al., 2021). This finding indicates the importance of a large sample size for effectively detecting and subsequently validating the discovered QTLs. Heritability is also one of the most important factors determining the efficiency of GWAS, with higher heritability usually detecting more SNPs for the same trait (Korte and Farlow, 2013; Wray et al., 2013). In this study, GWAS detected and validated more associated SNPs in WCW than in JW and MW, which may be related to the generally higher heritabilities of traits from WCW than from JW and MW (Figure 3A). GWAS also detected the largest number of associations with traits from EW and the lowest number of associations with traits from LW. This is consistent with the results reported in white spruce (Wray et al., 2013).

## Data availability statement

The raw data for exome capture presented in this study can be found in online repositories: (https://www.ncbi.nlm.nih.gov/ PRJNA731384).

## Author contributions

HW and Z-QC conceived and designed the study and wrote the manuscript. Z-QC and BK attended data collection. Z-QC performed data analyses. All the authors including HT and MG-G edited the manuscript. All authors contributed to the article and approved the submitted version.

## Funding

Funding was received from the Swedish Foundation for Strategic Research (SSF) (Grant Number: RBP14-0040). Z-QC was partly supported by the European Union Horizon 2020 research and innovation program under Grant No. 773383 (B4EST project).

## Conflict of interest

The authors declare that the research was conducted in the absence of any commercial or financial relationships that could be construed as a potential conflict of interest.

## Publisher's note

All claims expressed in this article are solely those of the authors and do not necessarily represent those of their affiliated organizations, or those of the publisher, the editors and the reviewers. Any product that may be evaluated in this article, or claim that may be made by its manufacturer, is not guaranteed or endorsed by the publisher.
