# Supplementary material for: Genetic architecture behind developmental and seasonal control of tree growth and wood properties in Norway spruce
Source: Front Plant Sci. 2022 Aug 9;13:927673. doi: 10.3389/fpls.2022.927673 (PMC9396349; doi:10.3389/fpls.2022.927673)
Supplement: Supplementary file 1 [file Data_Sheet_1.docx]

**Supplementary Figures**

**Figure S1 |**  Diagram of the development of plant materials for previous and current studies.

**Figure S2 |** Distribution of the number of rings per sample in the present study. Distribution of the number of rings per sample for 1303 unrelated progenies selected from 1303 half-sib families in the Höreda plantation (batch 1, 505 increment cores sampled in 2010 plus batch 2, 798 wood discs sampled in 2015), which results in the two peaks for this distribution.

**Figure S3 |** Boxplot of inflation factors estimated from the 267 GWAS runs. Those GWAS runs include 34 GWAS runs for each of three wood types (juvenile wood, mature wood, and whole wood) and also 34 GWAS runs for each of three methods (BLINK, univariate GEMMA (UV-GEMMA), and multivariate GEMMA (MV-GEMMA) GWAS).

**Figure S4 |** Pairwise Pearson phenotypic correlations of traits from juvenile wood (JW) measured in *Picea abies* field plantation, Sweden. The colour spectrum, bright blue to bright red represents highly positive to highly negative correlations and the number represents the correlation values. EW, TW, LW represent earlywood, transition wood, and latewood, respectively.

**Figure S5 |** Pairwise Pearson phenotypic correlations of traits from mature wood (MW) measured in *Picea abies* field plantation, Sweden. The colour spectrum, bright red to bright blue represents highly positive to highly negative correlations and the number represents the correlation values. EW, TW, LW represent earlywood, transition wood, and latewood, respectively.

**Figure S6 |** Manhattan plots comparing GEMMA univariate (UV-GEMMA), multivariate (MV-GEMMA), and BLINK GWAS in the whole core wood (WCW) of *Picea abies*. The colours of the dots correspond to univariate or multivariate associated. *P*-values are converted to –log_10_ (*P*-value). Single nucleotide polymorphisms (SNPs) above the red lines passed the Bonferroni correction test (*P*<3.7×10^-7^). SNPs above the blue line passed False Discovery Rate (FDR) at *P*<0.05 for the multivariate set in Figure S5a. Only SNPs with *P*<1*10^-2^ are plotted. a) multivariate set: wood density (annual ring wood, earlywood, transition wood, latewood) in whole core wood (WCW) using MV-GEMMA and UV-GEMMA. b) The same traits as a) using univariate model BLINK. c) linkage disequilibrium (LD) between SNPs associated with the multivariate set. The dashed line is to identify if MV-GEMMA or BLINK increase the power in the same contig as UV-GEMMA.

**Figure S7 |** QQ-plot for GWAS in Figure 6a and 6b in *Picea abies.* QQ-plot for univariate GWAS of transition wood (TW) ring width (a), TW density (b), TW coarseness (c), TW no. of cells (d), the corresponding multivariate GEMMA (MV-GEMMA) (e), univariate BLINK for TW ring width (f), TW density (g), TW coarseness (h), and TW no. of cells (i). IF is the inflation factor (if IF is within±0.05, we usually don’t consider population structure in GWAS model in this study). The red line is the fitted IF line. For corresponding Manhattan plots, see Figure 6a and 6b.

**Figure S8 |** QQ-plot for GWAS in Figure 6d and e in *Picea abies*. QQ-plot for univariate GWAS of no. of cells (a), ring width (b), radial tracheid width (c), the corresponding multivariate GEMMA (MV-GEMMA) (d), univariate BLINK for no. of cells (e), ring width (g), and radial tracheid width (h). IF is the inflation factor (if IF is within±0.05, we usually don’t consider population structure in GWAS model in the present study). The red line is the fitted IF line. For corresponding Manhattan plots, see Figure 6d and e.

**Figure S9 |** QQ-plot for GWAS in Figure S6a and b in *Picea abies.* QQ-plot for univariate GWAS of earlywood density (a), transition wood density (b), latewood density (c), the corresponding multivariate GEMMA (MV-GEMMA) (d), univariate BLINK for earlywood density(e), transition wood density (g), latewood density(h), and mean wood density (i). IF is the inflation factor (if IF is within±0.05, we usually don’t consider population structure in GWAS model in the present study). The red line is the fitted IF line. For corresponding Manhattan plots, see Figure S6a and b.


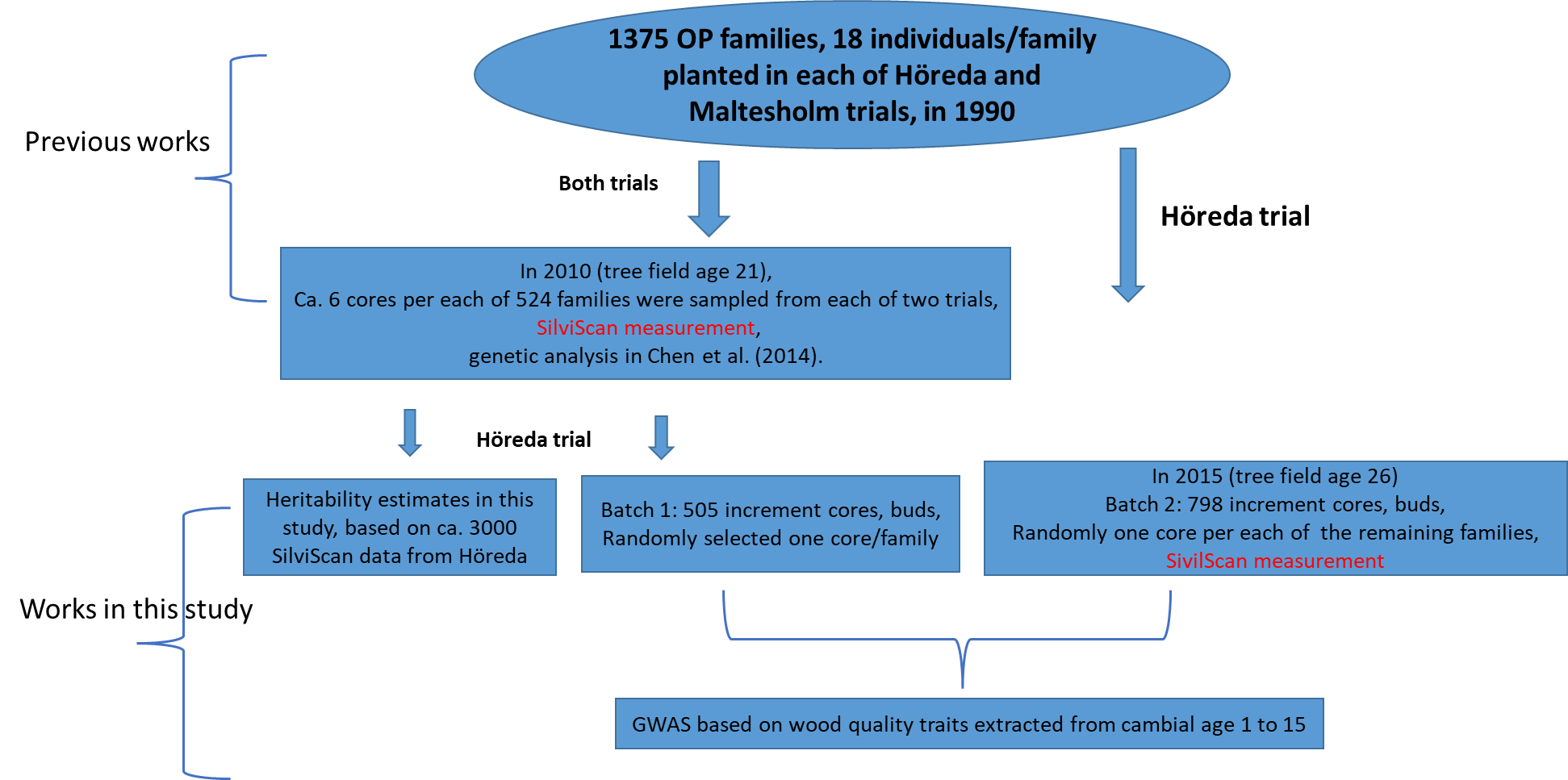


Figure S1. Diagram of the development of plant materials for previous and current studies.


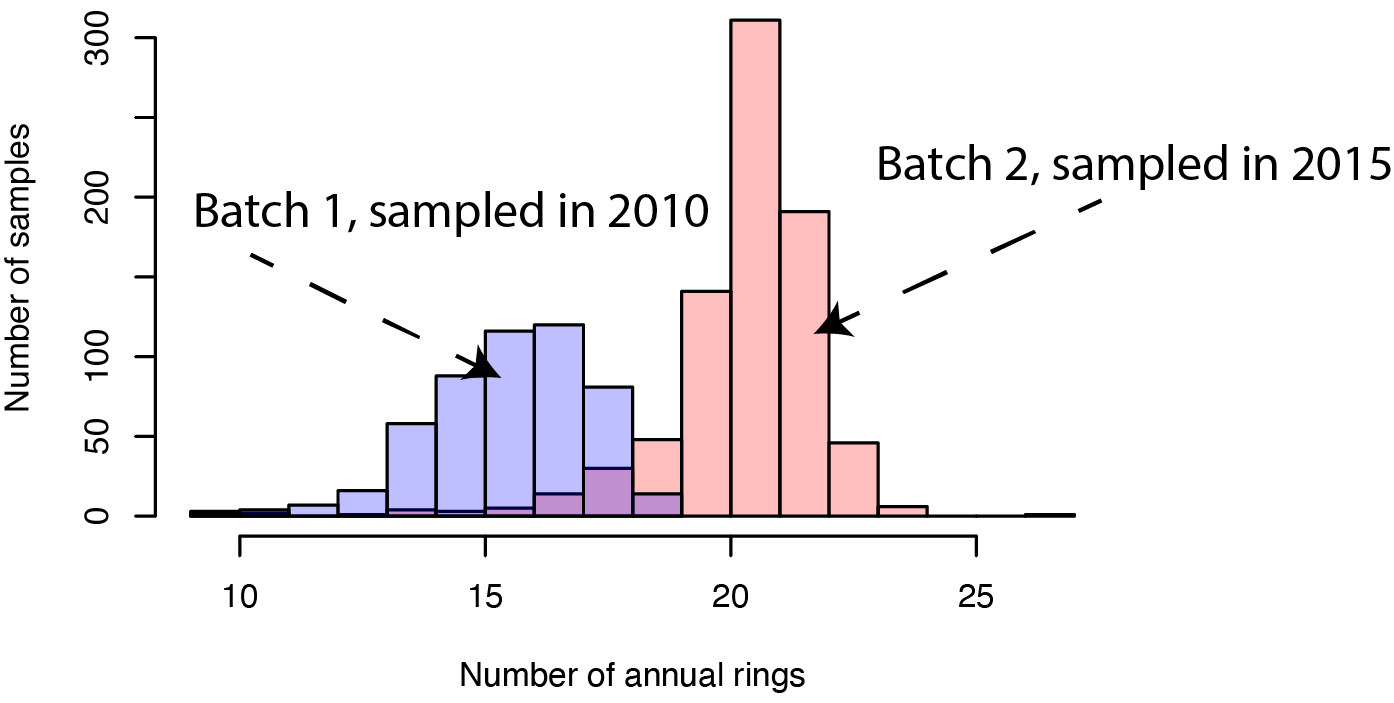


**Figure S2 |** Distribution of the number of rings per sample in the present study. Distribution of the number of rings per sample for 1303 unrelated progenies selected from 1303 half-sib families in the Höreda plantation (batch 1, 505 increment cores sampled in 2010 plus batch 2, 798 wood discs sampled in 2015), which results in the two peaks for this distribution.


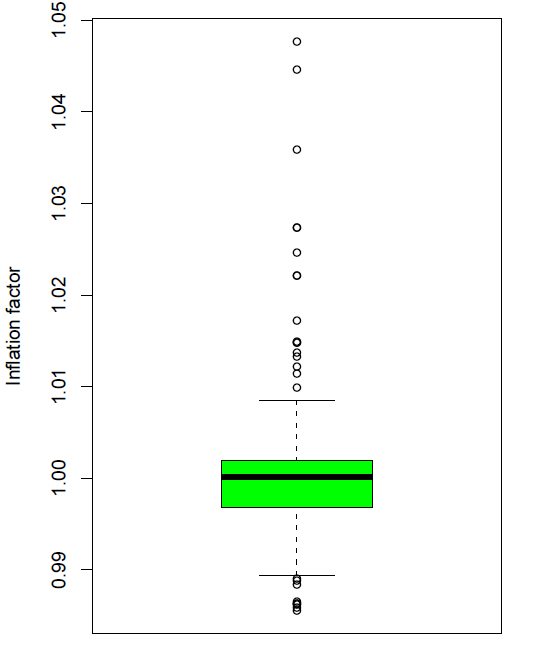


**Figure S3 |** Boxplot of inflation factors estimated from the 267 GWAS runs. Those GWAS runs include 34 GWAS runs for each of three wood types (juvenile wood, mature wood, and whole wood) and also 34 GWAS runs for each of three methods (BLINK, univariate GEMMA (UV-GEMMA), and multivariate GEMMA (MV-GEMMA) GWAS).


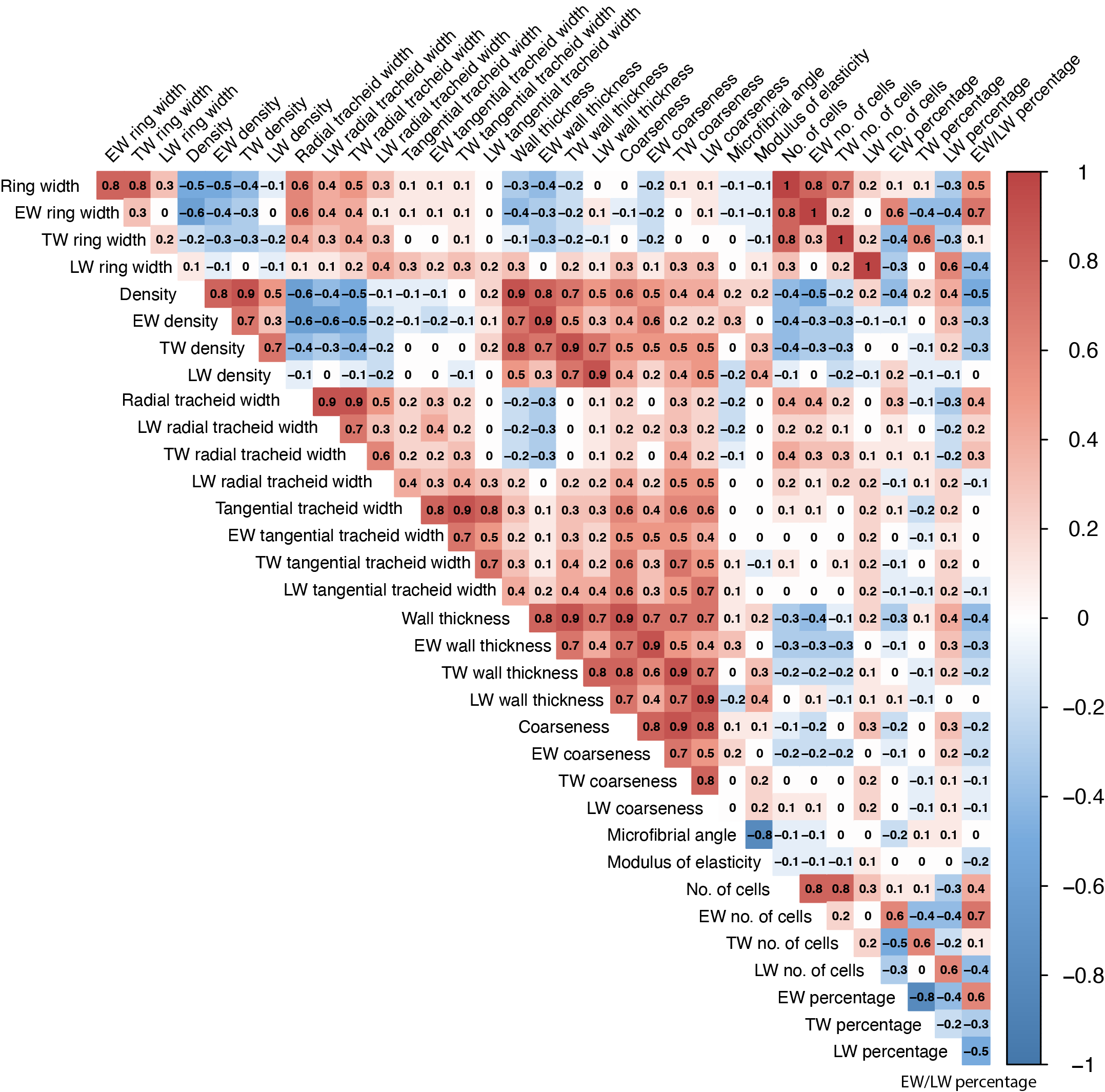


**Figure S4 |** Pairwise Pearson phenotypic correlations of traits from juvenile wood (JW) measured in *Picea abies* field plantation, Sweden. The colour spectrum, bright blue to bright red represents highly positive to highly negative correlations and the number represents the correlation values. EW, TW, LW represent earlywood, transition wood, and latewood, respectively.


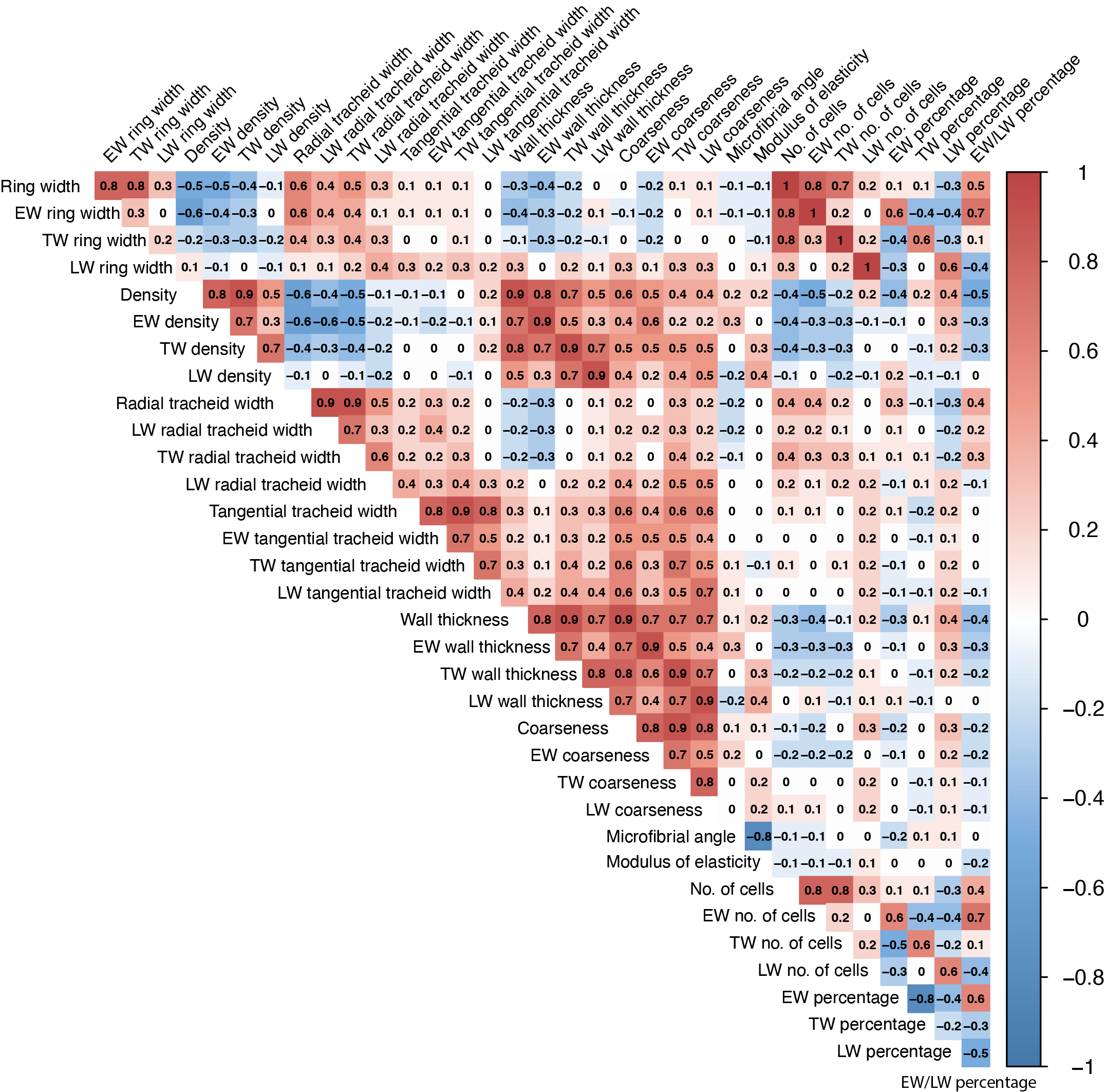


**Figure S5 |**  Pairwise Pearson phenotypic correlations of traits from mature wood (MW) measured in *Picea abies* field plantation, Sweden. The colour spectrum, bright red to bright blue represents highly positive to highly negative correlations and the number represents the correlation values. EW, TW, LW represent earlywood, transition wood, and latewood, respectively.


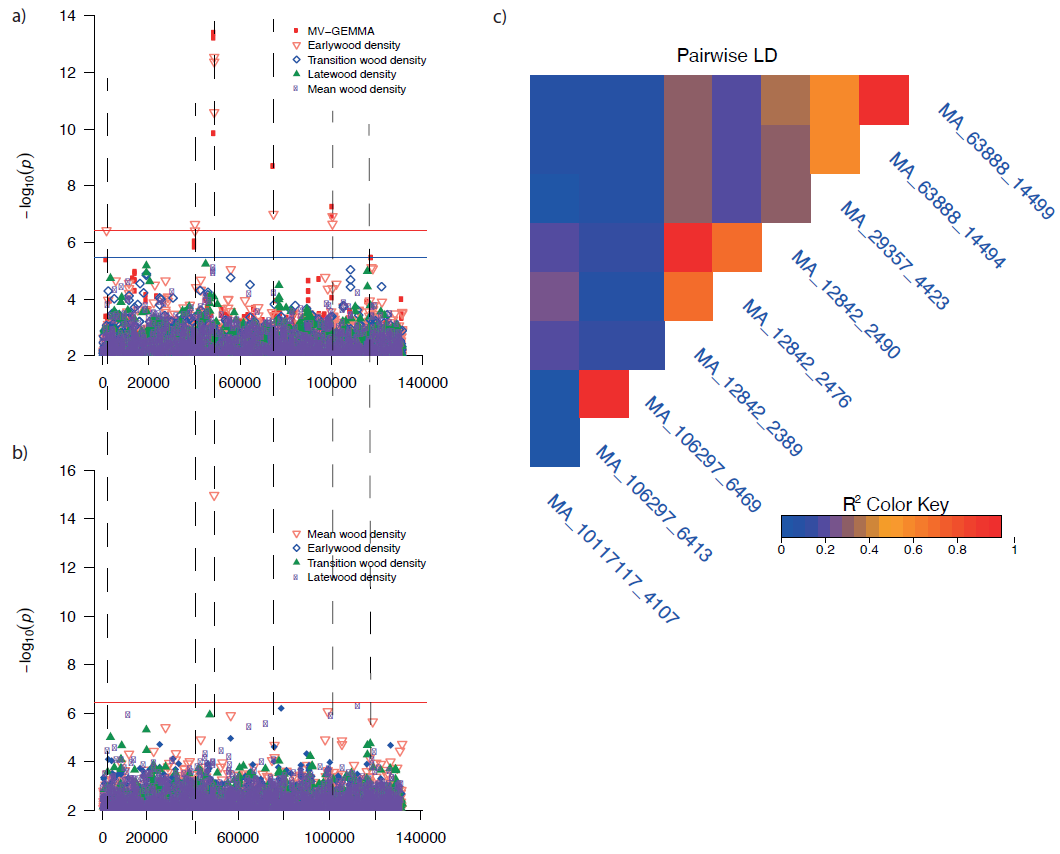


**Figure S6 |** Manhattan plots comparing GEMMA univariate (UV-GEMMA), multivariate (MV-GEMMA), and BLINK GWAS in the whole core wood (WCW) of *Picea abies*. The colours of the dots correspond to univariate or multivariate associated. *P*-values are converted to –log_10_ (*P*-value). Single nucleotide polymorphisms (SNPs) above the red lines passed the Bonferroni correction test (*P*<3.7×10^-7^). SNPs above the blue line passed False Discovery Rate (FDR) at *P*<0.05 for the multivariate set in Figure S5a. Only SNPs with *P*<1*10^-2^ are plotted. a) multivariate set: wood density (annual ring wood, earlywood, transition wood, latewood) in whole core wood (WCW) using MV-GEMMA and UV-GEMMA. b) The same traits as a) using univariate model BLINK. c) linkage disequilibrium (LD) between SNPs associated with the multivariate set. The dashed line is to identify if MV-GEMMA or BLINK increase the power in the same contig as UV-GEMMA.


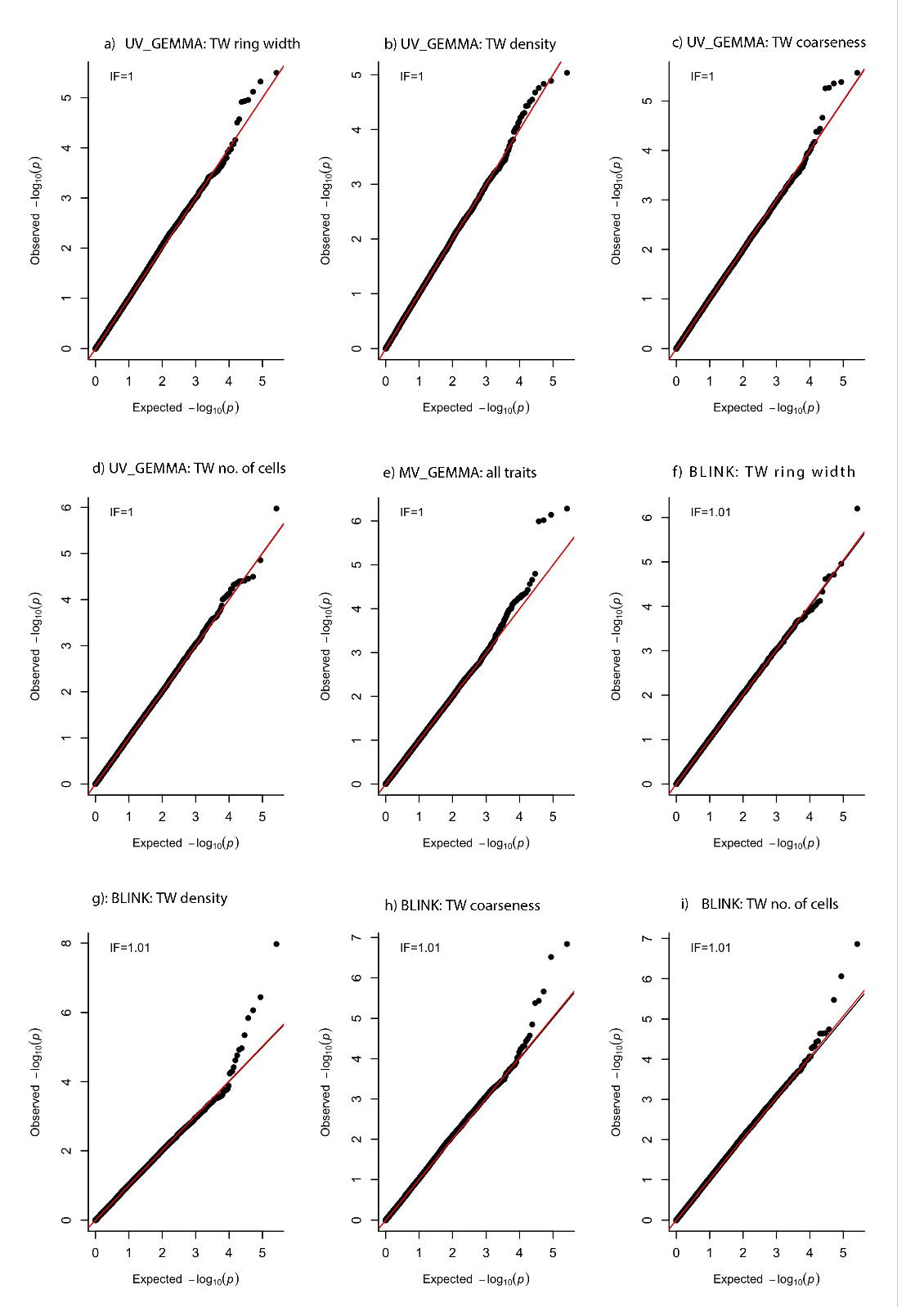


**Figure S7 |**  QQ-plot for GWAS in Figure 6a and b in *Picea abies.* QQ-plot for univariate GWAS of transition wood (TW) ring width (a), TW density (b), TW coarseness (c), TW no. of cells (d), the corresponding multivariate GEMMA (MV-GEMMA) (e), univariate BLINK for TW ring width (f), TW density (g), TW coarseness (h), and TW no. of cells (i). IF is the inflation factor (if IF is within±0.05, we usually don’t consider population structure in GWAS model in this study). The red line is the fitted IF line. For corresponding Manhattan plots, see Figure 6a and b.


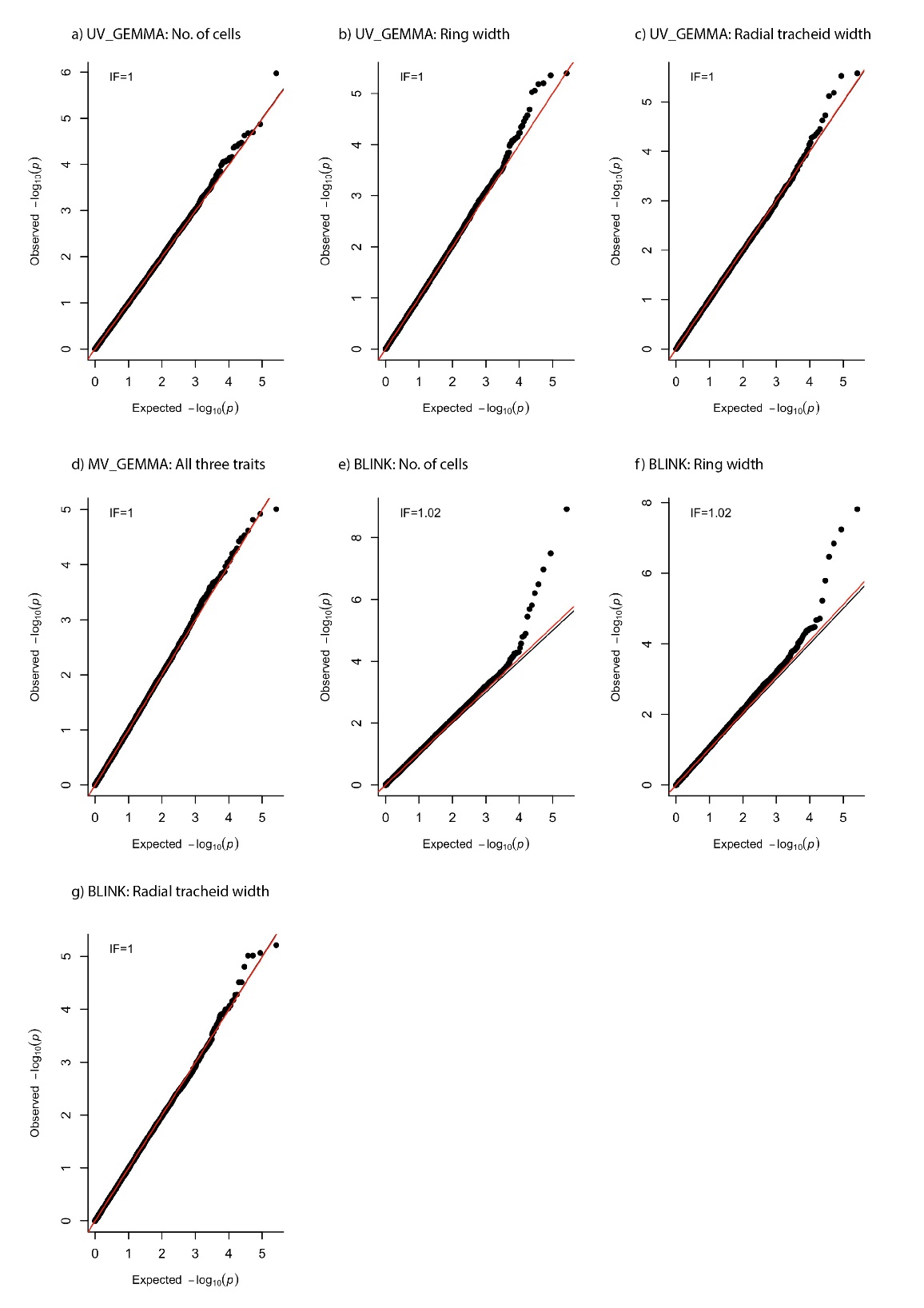


**Figure S8 |** QQ-plot for GWAS in Figure 6d and e in *Picea abies*. QQ-plot for univariate GWAS of no. of cells (a), ring width (b), radial tracheid width (c), the corresponding multivariate GEMMA (MV-GEMMA) (d), , univariate BLINK for no. of cells €, ring width (f), and radial tracheid width (g). IF is the inflation factor (if IF is within±0.05, we usually don’t consider population structure in GWAS model in the present study). The red line is the fitted IF line. For corresponding Manhattan plots, see Figure 6d and e.


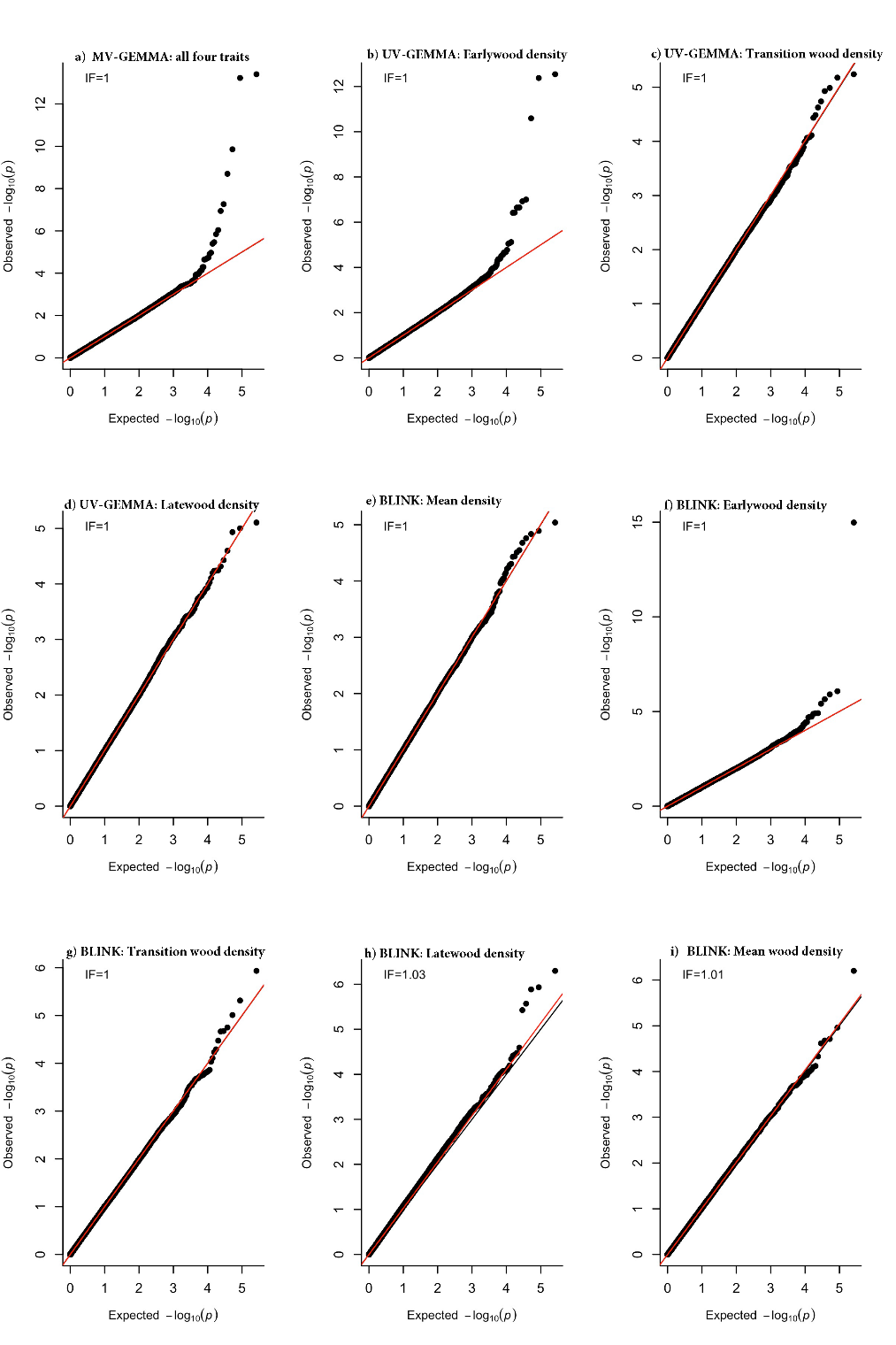


**Figure S9 |** QQ-plot for GWAS in Figure S6a and b in *Picea abies.* QQ-plot for the corresponding multivariate GEMMA (MV-GEMMA) (a), univariate GWAS of earlywood density (b), transition wood density (c), latewood density (d), , univariate BLINK for earlywood density(e), transition wood density (g), latewood density(h), and mean wood density (i). IF is the inflation factor (if IF is within±0.05, we usually don’t consider population structure in GWAS model in the present study). The red line is the fitted IF line. For corresponding Manhattan plots, see Figure S6a and b.
