# Supplementary material for: Genetic architecture behind developmental and seasonal control of tree growth and wood properties in Norway spruce
Source: Front Plant Sci. 2022 Aug 9;13:927673. doi: 10.3389/fpls.2022.927673 (PMC9396349; doi:10.3389/fpls.2022.927673)
Supplement: Supplementary file 3 [file Table_2.DOCX]

**Supplementary**

**Methods S1 | Methods to get wood coarseness, wood stiffness, and number of cells**

Wood coarseness was predicted with predictor traits of radial tracheid, tangential tracheid, and WD according to a formula wood coarseness = radial tracheid width × tangential tracheid width × WD (Ma *et al.*, 2018). Wood stiffness, expressed as modulus of elasticity (MOE), was predicted using the predictor traits MFA and WD according to a formula (Evans & Elic, 2001). Number of cells was predicted using the predictor traits ring width and radial tracheid width according to a formula (number of cells = ring width ÷ radial tracheid width).

**Methods S2 | SNP calling**

Raw reads based on 2×100 bp sequencing mode were mapped to the *P. abies* reference genome v1.0 using BWA-mem (Langmead & Salzberg, 2012). SAMTools (Li *et al.*, 2009) and Picard (McKenna *et al.*, 2010) were used for sorting and removal of PCR duplicates, and the resulting BAM files were subsequently reduced to include only probe bearing scaffolds (24919) before variant calling. Variant calling was performed using Genome Analysis Toolkit (GATK) HaplotypeCaller (McKenna *et al.*, 2010) in Genome Variant Call Format (gVCF) output format.

**Methods S3 | SNP quality control**

The following seven steps were used to perform SNP quality control using VCFtools.

(1) removing indels, (2) keeping only bi-allelic sites, (3) marking sites with a genotype quality (GQ) < 10 as missing, (4) removing sites with call rate < 70%, (5) removing sites with minor allele frequency (MAF) < 0.005, (6) removing sites with an excess of heterozygotes and deviation from Hardy-Weinberg equilibrium test (HWE-test) for unrelated population (*p*-value < 1.4×10e-7), and (7) removing individuals with call rate < 50%.

**Methods S4 | Adjustment of phenotypic data**

The phenotypic SilviScan data from 1303 trees was adjusted using the following equation:

$\boldsymbol{y=X}b\boldsymbol{+Z}u\boldsymbol{+}\epsilon$ **[1]**

Where *y* is the vector with tree height, DBH, and one of the 34 SilviScan traits in each of three trait types (Table 1), *b* is the vector of fixed effects including an intercept for three traits, *u* is the vector of random effects including post-block, incomplete block, and additive effects. ***X*** and ***Z*** are the corresponding design matrices of *b* and *u*, $\epsilon$ is the vector of residuals for the three traits. An unstructured covariance was used in the model fitting for each of the random terms. The phenotypic values entering the GWAS were the original phenotypic data adjusted for the post-block and randomized incomplete block effects.

**Methods S5 | Estimate of narrow-sense heritability**

Pedigree-based narrow-sense heritability ($h_{a}^{2}$) and SNP-based narrow-sense heritability ($h_{g}^{2})$ were calculated as:

$h_{a}^{2}= \frac{\sigma_{a}^{2}}{\sigma_{pa}^{2}}$ **[2]**

$h_{g}^{2}= \frac{\sigma_{g}^{2}}{\sigma_{pg}^{2}}$ **[3]**

respectively, where $\sigma_{a}^{2}$ and $\sigma_{g}^{2}$ are additive variance based on pedigree and genomic marker models, respectively. $\sigma_{pa}^{2}$ and $\sigma_{pg}^{2}$ are phenotypic variances for pedigree-based and marker-based models, respectively.

**Methods S6 | BLINK method**

In this study, we performed three Genome-wide association (GWA) methods. BLINK algorithm is developed by Huang et al. (2019) (Huang et al., 2019). Here we slightly modified the definition for the algorithm as below:

The BLINK method conducts two fixed effect models and one filtering process, which selects a set of pseudo quantitative trait nucleotides (QTNs) that are not in LD with each other as covariates. The entire sequence runs repeatedly until all genetic markers are tested and the selection of pseudo-QTNs is optimized. The first fixed-effect model tests *M* genetic markers, one at a time. Pseudo QTNs are included as covariates to simultaneously control false positives and reduce false negatives. Specifically, the first fixed-effect model can be written as follows:

$y_{i}=S_{i1}^{*}b_{1}+S_{i1}^{*}b_{2}+\ldots+S_{ik}^{*}b_{k}+S_{ij}^{*}d_{j}+e_{i}$…………………………………………………………[5]

where yi is the observation on the ith individual; $S_{i1}$, $S_{i2}$, . . . , $S_{ik}$ are the genotypes of k pseudo QTNs, initiated as an empty set; b_1_, b_2_, . . . , bk are the corresponding effects of the pseudo QTNs; $S_{ij}$ is the genotype of the ith individual and jth genetic marker; dj is the corresponding effect of the jth genetic marker; and $e_{i}$ is the residual having a distribution with a mean of zero and a variance of $\sigma_{e}^{2}$.

The primary goal of the first fixed effect model is to calculate the *P* values for all *M* testing markers. The second FEM is employed to optimize the selection of pseudo-QTNs. Specifically, the second FEM can be written as follows:

$yi=S_{i1}^{*}b_{1}+s_{i2}^{*}b_{2}+\ldots+s_{ik}^{*}b_{k}+e_{i}$ …………………………………………………………… [6]

Equations [5] and [6] differ in two ways. First, the testing marker term in the first FEM is removed from the second FEM; therefore, no testing marker *P* values are output in equation [6]. Second, the number of covariate pseudo-QTNs is varied in the second FEM to select the optimum set of the first k out of t pseudo QTNs. The optimization is performed using BIC, which is twice the negative log-likelihood plus the penalty on the number of parameters, as follows:

$BIC = -2LL + 2kLn (n)$ ………………………………………………………………………………[7]

where LL is the log-likelihood, k is the number of pseudo QTNs, Ln is the natural log, and n is the number of individuals. The available pseudo QTNs, t, are sorted with the most significant at the beginning and the least significant at the end. The first k pseudo QTNs are selected for examination, with k varied from 1 to t.

All markers in equation [5] are candidates for pseudo QTNs in equation [6]. These markers are filtered with two criteria: *P*-value and correlation. All markers are sorted first and then filtered out if their *P* values are larger than a threshold (Bonferroni correction, *α* = 0.01). Of the m SNPs remaining, if their correlation, *r* (Pearson correlation), with the first SNP ($S_{1}^{*}$) is larger than a threshold (0.7), they are also removed. This process is repeated to select $S_{2}^{*}$, $S_{3}^{*}$, . . . , until the last SNP, $S_{t}^{*}$), is selected.

Because the t remaining markers are sorted and not highly correlated with each other, the first set of k markers is more critical than the second set of k markers. We fit the first k markers in equation [6] and vary k until all possibilities are examined. The set of k markers with the best BIC is used as the set of pseudo QTNs in equation [5]. This process is iterated until the pseudo-QTNs remain the same.

This alternative solution is called the Bayesian-information and linkage-disequilibrium iteratively nested keyway (BLINK) method.

**Methods S7 |** Multi-variate GEMMA (MV-GEMMA) model:

A multivariate linear mixed model was used in GEMMA software (Zhou & Stephens, 2014) as in the following form:

$$Y = \mathrm{WA} + x\beta^{T} + U + E; G\sim\mathrm{MN}_{n\times d}\left( 0, K,V_{g} \right), E\sim\mathrm{MN}_{n\times d}\left( 0,I_{n\times n}, V_{e} \right),$$

where Y is an *n* by *d* matrix of *d* phenotypes for n individuals; W = ($w_{1}$, · · · , w_c_) is an *n*×*c* matrix of covariates (fixed effects) including a column of 1s; A is a *c* by *d* matrix of the corresponding coefficients including the intercept; x is an *n*-vector of marker genotypes; *β* is a *d* vector of marker effect sizes for the *d* phenotypes; U is an *n* by *d* matrix of random effects; E is an *n* by d matrix of errors; K is a known *n* by *n* relatedness matrix, I*_n_*_×_*_n_* is a *n* by *n* identity matrix, **V**_g_ is a *d* by *d* symmetric matrix of genetic variance component, V_e_ is a *d* by *d* symmetric matrix of environmental variance component and MN_n×d_(0, V_1_, V_2_) denotes the *n*×*d* matrix normal distribution with mean 0, row covariance matrix V_1_ (*n* by *n*), and column covariance matrix V_2_ (*d* by *d*).

**Methods S8 | Estimate of the cumulative proportion of phenotypic variance explained (PVE).**

In the estimate of cumulative PVE by all candidate SNPs for each trait, variance components of random effects were calculated from the following two equations:

$\boldsymbol{y=W}\alpha_{1}\boldsymbol{+X}\beta_{1}+\boldsymbol{\epsilon}_{\boldsymbol{1}}$…………………………………………………………………………[8]

and

$\boldsymbol{y=W}\alpha_{2}\boldsymbol{+Zs+X}\beta_{2}+\boldsymbol{\epsilon}_{\boldsymbol{2}}$……………………………………………………………………[9]

where y is a vector of adjusted phenotypic values, $\alpha_{1}$ and $\alpha_{1}$ are two vectors of corresponding fixed effects including the intercept in equations [8] and [9], respectively. $\beta_{1}$ and $\beta_{2}$ are two vectors of random additive genetic effects in equations [8] and [9], respectively, following $\beta_{1}\sim N(\mathbf{0},G\sigma_{a1}^{2})$ and $\beta_{2}\sim N(\mathbf{0},G\sigma_{a2}^{2})$. *G* is a genomic-based relationship matrix (GRM) based on all SNPs (~134K). $\sigma_{a1}^{2}$ and $\sigma_{a2}^{2}$ are the additive variances in equations [8] and [9], respectively. ***s*** is a vector of all candidate SNPs as fixed effects for each trait. $\epsilon_{1}$ and $\epsilon_{2}$ are two vectors of residual effects ~ N(0,$\sigma_{e1}^{2}$I) and ~ N(0,$\sigma_{e2}^{2}I$) in equations [8] and [9], respectively. $\mathbf{0}$ is a null vector. *Z*, *W*, and *X* are the related design matrices in both equations:

$Cumulative PVE = \frac{\sigma_{a1}^{2}+\sigma_{e1}^{2}-\sigma_{a2}^{2}-\sigma_{e2}^{2}}{\sigma_{a1}^{2}+\sigma_{e1}^{2}} \times100$ …………………………………………………[10]

Note: if in some of the cases, the numerator of equation [7] is less than zero, we report it as zero as a percentage of variance explained by an SNP or SNPs could not be less than zero. This also represents that if a genomic prediction model includes the SNP or SNPs as a fixed effect or effects, the prediction model will not be improved in a real case.

**Methods S9 | Validation test for candidate SNP detected by GWAS**

Using ASReml R v4.0, a Wald test was used to test the significance of candidate SNP for each trait as below:

$\boldsymbol{y=W}\alpha\boldsymbol{+Zs+X}\beta+\boldsymbol{\epsilon}$ ………………………………………………………………………[12]

where y is the vector of adjusted phenotypic values, $\alpha$ is a vector of corresponding fixed effect including the intercept, $\beta$ is a vector of random additive genetic effects, following $\beta\sim N(\mathbf{0},G\sigma_{a}^{2})$. *G* is a genomic-based relationship matrix (GRM) based on all SNPs (~134K). $\sigma_{a}^{2}$ is the additive variance. s is a single candidate SNP effect. $\epsilon$ is a vector of residuals. $\mathbf{0}$ is a null vector. *Z*, *W*, and *X* are the related design matrices.

**References**:

**Evans R, Elic J. 2001.** Rapid prediction of wood stiffness from microfibril angle and density. *Forest Products Journal* **51**(3): 53–57.

**Huang M, Liu X, Zhou Y, Summers RM, Zhang Z. 2019.** BLINK: a package for the next level of genome-wide association studies with both individuals and markers in the millions. *Gigascience* **8**(2): 1–12.

**Langmead B, Salzberg SL. 2012.** Fast gapped-read alignment with Bowtie 2. *Nature Methods* **9**: 357–359.

**Li H, Handsaker B, Wysoker A, Fennell T, Ruan J, Homer N, Marth G, Abecasis G, Durbin R. 2009.** The sequence alignment/map format and SAMtools. *Bioinformatics* **25**(16): 2078−2079.

**Ma T, Inagaki T, Tsuchikawa S. 2018.** Non-destructive evaluation of wood stiffness and fiber coarseness, derived from SilviScan data, via near infrared hyperspectral imaging. *Journal of Near Infrared Spectroscopy* **26**(6): 398-405.

**McKenna A, Hanna M, Banks E, Sivachenko A, Cibulskis K, Kernytsky A, Garimella K, Altshuler D, Gabriel S, Daly M, et al. 2010.** The Genome Analysis Toolkit: A MapReduce framework for analyzing next-generation DNA sequencing data. *Genome Research* **20**(9): 1297−1303.

**Zhou X, Stephens M. 2014.** Efficient multivariate linear mixed model algorithms for genome-wide association studies. *Nature Methods* **11**(4): 407–409.
